# Assembly and comparative analysis of the complete mitochondrial genome of *Ilex metabaptista* (Aquifoliaceae), a Chinese endemic species with a narrow distribution

**DOI:** 10.1186/s12870-023-04377-7

**Published:** 2023-08-14

**Authors:** Peng Zhou, Qiang Zhang, Fei Li, Jing Huang, Min Zhang

**Affiliations:** 1 Jiangsu Academy of Forestry, 109 Danyang Road, Dongshanqiao Nanjing, 211153 China; 2 Co-Innovation Center for Sustainable Forestry in Southern China, Key Laboratory of State Forestry and Grassland Administration on Subtropical Forest Biodiversity Conservation, College of Biology and the Environment, Nanjing Forestry University, 210037 Nanjing, China

**Keywords:** *Ilex metabaptista*, Mitochondrial genome, Comparative analysis, Phylogenetic analysis, Repeat sequence

## Abstract

**Background:**

*Ilex metabaptista* is a woody tree species with strong waterlogging tolerance and is also admired as a landscape plant with high development prospects and scientific research value. Unfortunately, populations of this species have declined due to habitat loss. Thus, it is a great challenge for us to efficiently protect *I. metabaptista* resources from extinction. Molecular biology research can provide the scientific basis for the conservation of species. However, the study of *I. metabaptista* genetics is still in its infancy. To date, no mitochondrial genome (mitogenome) in the genus *Ilex* has been analysed in detail.

**Results:**

The mitogenome of *I. metabaptista* was assembled based on the reads from Illumina and Nanopore sequencing platforms; it was a typical circular DNA molecule of 529,560 bp with a GC content of 45.61% and contained 67 genes, including 42 protein-coding genes, 22 tRNA genes, and 3 rRNA genes. Repeat sequence analysis and prediction of RNA editing sites revealed a total of 286 dispersed repeats, 140 simple repeats, 18 tandem repeats, and 543 RNA editing sites. Analysis of codon usage showed that codons ending in A/T were preferred. Gene migration was observed to occur between the mitogenome and chloroplast genome via the detection of homologous fragments. In addition, Ka/Ks analysis revealed that most of the protein-coding genes in the mitogenome had undergone negative selection, and only the *ccmB* gene had undergone potential positive selection in most asterids. Nucleotide polymorphism analysis revealed the variation in each gene, with *atp9* being the most notable. Furthermore, comparative analysis showed that the GC contents were conserved, but the sizes and structure of mitogenomes varied greatly among asterids. Phylogenetic analysis based on the mitogenomes reflected the exact evolutionary and taxonomic status of *I. metabaptista*.

**Conclusion:**

In this study, we sequenced and annotated the mitogenome of *I. metabaptista* and compared it with the mitogenomes of other asterids, which provided essential background information for further understanding of the genetics of this plant and helped lay the foundation for future studies on molecular breeding of *I. metabaptista.*

**Supplementary Information:**

The online version contains supplementary material available at 10.1186/s12870-023-04377-7.

## Background

*Ilex* L. (holly), from the monogeneric family Aquifoliaceae, is one of the largest woody dioecious angiosperm genera, and it contains approximately 600 species widely distributed from the tropics to temperate regions [1]. As an evergreen shrub, *I. metabaptista* Loes. ex Diels grows beside the river beach at altitudes of 300–1200 m and is only found in Chongqing, Guangxi, Guizhou, Hubei, Hunan and Sichuan in China [2]. It displays a strong waterlogging tolerance capacity and has high horticultural value [3]. As a valuable endemic species with small populations, it is regarded as a natural resource with potential economic and ecological importance. Unfortunately, populations of this species have declined due to continuing declines in the area and extent of habitat [4]. Thus, it is a great challenge to efficiently protect *I. metabaptista* resources from extinction. The investigation of the molecular diversity and evolution of this species will help establish more effective conservation countermeasures for the future [5]. However, there has been little progress in the industrial development of *I. metabaptista* for a long time due to a lack of genomic resources and unclear genetic relationships.

Mitochondria and chloroplasts are organelles with a semiautonomous genetic system in higher plant cells, and they carry relevant genetic information [6, 7]. The nuclear genomes carry the overwhelming majority of information, but the chloroplast and mitochondrial genomes are nonetheless also indispensable in eukaryotes [8]. The plant mitogenomes have undergone rapid and tremendous structural changes since the initial endosymbiotic event [9–11]. Thus, the mitogenomes of plants are approximately 100 − 10,000 times larger and more structurally complex than those of animals [12]. The mitogenomes of land plants demonstrate large genome size variation, ranging from 66 kb in *Viscum scurruloideum* [13] to 11.7 Mb in *Larix sibirica* [14], which can be attributed to the frequent recombination of repetitive sequences and incorporation of foreign sequences via intracellular or horizontal transfer [9, 15]. The number of genes in land plant mitogenomes varies widely, typically between 32 and 67 [16, 17]; however, the functional genes exhibit substantial conservation [9, 11]. Additionally, structural complexity is another important feature of plant mitogenomes. Although plant mitogenomes have low mutation rates when compared to plastid (3–5 times lower) and nuclear genomes (10–20 times lower), the structures and gene orders are highly variable in plants [17–20].

Mitochondria are a powerful tool for studying the origin of species, genetic diversity, and phylogenetics [12, 21]. However, it is difficult to purify plant mitochondria, which are often interfered with by chloroplasts and other plastids [15], and to assemble their genomes due to their complex structure [16, 22], which makes it comparatively challenging to carry out plant mitogenome studies. To date, more than 5000 plant chloroplast genomes have been sequenced, but only approximately 400 plant mitogenomes have been published in the NCBI database [12]. In addition, sequenced plants largely differ in their classification with a strong bias towards crops [23], and only one complete mitogenome of species from the order Aquifoliales has been identified [24]. Plant mitogenomes vary greatly in both genome structure and content, nucleotide substitution rates, and repeat recombination levels [18, 25]. These variations in mitogenomes are observed not only between plant species but also within the same species [12, 26], in stark contrast to the conserved structure of plant chloroplast genomes [22]. Thus, the mitogenome is a valuable source of genetic information for the study of plant phylogeny and essential cellular processes [6]. Furthermore, the mitogenome is widely used in evolutionary analysis and interspecies discrimination studies, especially for the construction of ancient phylogenetic relationships and those among close species, because its genetic system is typically inherited maternally, relatively independent of the nucleus and relatively conserved [15, 27–29].

To date, the complete chloroplast genome sequences of a total of 55 *Ilex* species have been made available in the NCBI GenBank database (accessed on 4 May 2023), and nuclear genome sequencing has been performed in *I. latifolia* [30], *I. asprella* [31], and *I. polyneura* [32]. To date, no mitogenome in the genus *Ilex*, except for the mitogenome sequence of *I. pubescens* released in 2019 [24], has been analysed in detail, which might greatly hinder a deep understanding of the evolution of mitogenomes in this large family. The complete chloroplast genome of *I. metabaptista* has already been assembled (GenBank Accession number: NC_069021.1); however, no report on the mitochondrial and nuclear genomes of this species has yet been published.

Therefore, in this study, the *I. metabaptista* mitogenome was sequenced and annotated for the first time. In addition, we conducted a comprehensive analysis with regard to genomic characteristics, repetitive sequences, RNA editing, codon preference, migration sequences and comparative genomics with other asterids and performed a phylogenetic analysis. These results will help better understand the structure and function of the *I. metabaptista* mitogenome and provide useful molecular markers for conservation biology, population genetics, and evolutionary studies on this species.

## Results

### Sequencing and genomic features of the *I. metabaptista* mitogenome

**Fig. 1:**
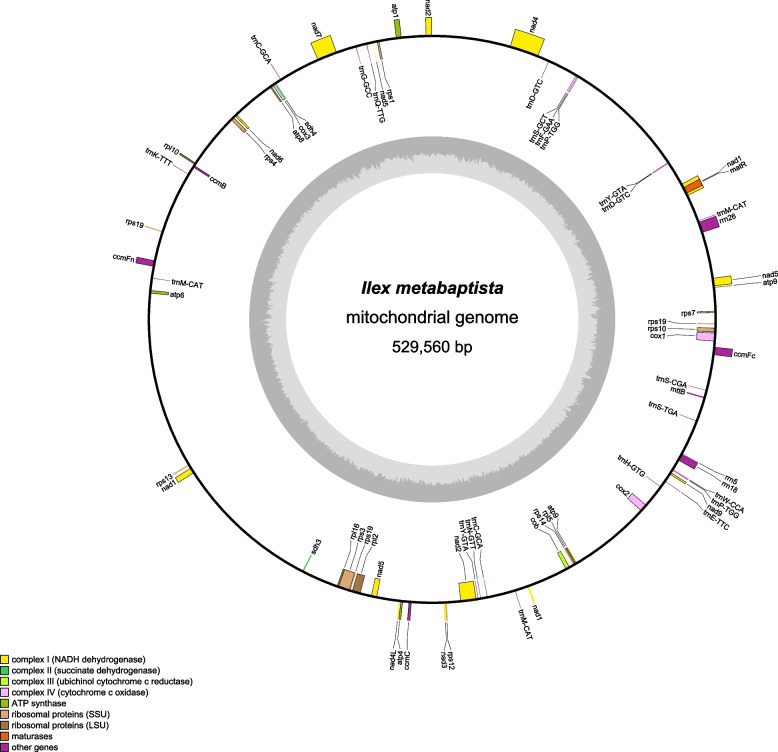
Circular map of the *I. metabaptista* mitogenome. Gene map showing 67 annotated genes of different functional groups. Genes shown on the outside and inside of the circle are transcribed clockwise and counterclockwise, respectively. The dark grey region in the inner circle depicts the GC content

The total DNA of *I. metabaptista* was sequenced, and the raw data were prepared for assembly, resulting in 12.45 G Illumina sequencing data and 14.41 G Nanopore PromethION sequencing data with an average read length of 8,863 bp (Table S1). We then assembled the complete mitogenome of *I. metabaptista*, which was a circular sequence with a length of 529,560 bp. The functional classifications and physical locations of the annotated genes are shown in Fig. 1. In the *I. metabaptista* mitogenome, 67 genes, including 42 protein-coding genes (PCGs), 22 tRNA genes, and 3 rRNA genes, were annotated. Additionally, 3,122 open reading frames (ORFs) were identified.

The *I. metabaptista* mitogenome encoded 39 different proteins (*atp9* has two copies, and *rps19* has three copies) that could be divided into 10 categories (Table 1): ATP synthases (5 genes), cytochrome C biogenesis (4 genes), ubiquinol cytochrome c reductases (1 gene), cytochrome C oxidases (3 genes), maturases (1 gene), transport membrane proteins (1 gene), NADH dehydrogenases (9 genes), ribosomal proteins (LSU; 4 genes), ribosomal proteins (SSU; 9 genes) and succinate dehydrogenase (2 genes).

**Table 1:** Functional classifications and physical locations of genes in the *I. metabaptista* mitogenome

Group of genes	Gene name	Length	Start codon	Stop codon	Amino acids
ATP synthase	*atp1*	1530	ATG	TAA	510
	*atp4*	579	ATG	TAA	193
	*atp6*	885	ATG	TAA	295
	*atp8*	480	ATG	TAA	160
	*atp9 (2)*	(225,225)	ATG	CGA(TGA)	75
Cytochrome c biogenesis	*ccmB*	621	ATG	TGA	207
	*ccmC*	753	ATG	TGA	251
	*ccmFc*	1317	ATG	CGA(TGA)	439
	*ccmFn*	1734	ATG	TGA	578
Ubiquinol cytochrome c reductase	*cob*	1182	ATG	TGA	394
Cytochrome c oxidase	*cox1*	1497	ATG	TAA	499
	*cox2*	783	ATG	TAA	261
	*cox3*	798	ATG	TGA	266
Maturases	*matR*	1971	ATG	TAG	657
Transport membrane protein	*mttB*	342	ATG	TAA	114
NADH dehydrogenase	*nad1*	978	ATG	TAA	326
	*nad2*	1467	ATG	TAA	489
	*nad3*	357	ATG	TAA	119
	*nad4*	1488	ATG	TGA	496
	*nad4L*	303	ACG(ATG)	TAA	101
	*nad5*	2013	ATG	TAA	671
	*nad6*	618	ATG	TAA	206
	*nad7*	1185	ATG	TAG	395
	*nad9*	573	ATG	TAA	191
Ribosomal proteins (LSU)	*rpl10*	489	ATG	TAA	163
	*rpl16*	516	ATG	TAA	172
	*rpl2*	1011	ATG	TAA	337
	*rpl5*	564	ATG	TAA	188
Ribosomal proteins (SSU)	*rps1*	513	ATG	TAA	171
	*rps10*	363	ACG(ATG)	TGA	121
	*rps12*	378	ATG	TGA	126
	*rps13*	351	ATG	TGA	117
	*rps14*	303	ATG	TAG	101
	*rps19 (3)*	(285,285,285)	ATG	TAA	95
	*rps3*	1692	ATG	TAG	564
	*rps4*	1047	TTG	TAA	349
	*rps7*	447	ATG	TAA	149
Succinate dehydrogenase	*sdh3*	303	ATG	TGA	101
	*sdh4*	387	ATG	CGA(TGA)	129
Ribosomal RNAs	*rrn18*	1931			
	*rrn26*	3198			
	*rrn5*	121			
Transfer RNAs	*trnC-GCA (2)*	(71,82)			
	*trnD-GTC (2)*	(74,74)			
	*trnE-TTC*	72			
	*trnF-GAA*	74			
	*trnG-GCC*	72			
	*trnH-GTG*	74			
	*trnK-TTT*	73			
	*trnM-CAT (3)*	(73,74,74)			
	*trnN-GTT*	72			
	*trnP-TGG (2)*	(74,75)			
	*trnQ-TTG*	72			
	*trnS-CGA*	58			
	*trnS-GCT*	88			
	*trnS-TGA*	87			
	*trnW-CCA*	74			
	*trnY-GTA (2)*	(69,83)			

Numbers after gene names are the number of copies

Studies have shown that the mitogenomes of most terrestrial plants contain 3 rRNA genes [33]. Here, 3 rRNA genes from the *I. metabaptista* mitogenome, namely, *rrn18* (1931 bp), *rrn26* (3198 bp), and *rrn5* (121 bp), were annotated. In addition, 16 different tRNA genes (*trnC-GCA, trnD-GTC, trnP-TGG*, and *trnY-GTA* had two copies, and *trnM-CAT* had three copies) were identified in the *I. metabaptista* mitogenome. The length of these tRNAs ranged from 58 to 88 bp, with a total length of 1,639 bp.

The length of all PCGs was 33,123 bp, accounting for only 6.25% of the total mitogenome length. There were 55 genes with no introns, accounting for 82.09% of the total. In addition, 26 introns were found in the other 12 *I. metabaptista* mitochondrial genes; *nad1*, *nad2*, *nad5*, and *nad7* had 4 introns; and *nad4* had 3 introns.

The nucleotide composition of the whole mitogenome (Table 2) was A (27.27%), T (27.12%), C (22.70%), and G (22.91%). The entire mitogenome had a GC content of 45.61%, composed of 43.18% PCGs, 51.83% rRNAs, and 50.82% tRNAs. Strikingly, the GC content of the PCGs was lower than that of other CDS regions (tRNAs and rRNAs). The GC skew was positive in CDS regions and in the mitogenome.

**Table 2:** Composition and skewness of the *I. metabaptista* mitogenome

	Size (bp)	A%	T%	C%	G%	A + T%	G + C%	AT-skew	GC-skew
Mitogenome	529,560	27.27	27.12	22.70	22.91	54.39	45.61	0.003	0.005
PCGs	33,123	26.54	30.28	21.29	21.89	56.82	43.18	-0.066	0.014
rRNAs	5,250	26.13	22.02	22.69	29.16	48.15	51.85	0.085	0.125
tRNAs	1,639	22.57	26.6	22.57	28.25	49.18	50.82	-0.082	0.112

### Repeat sequence analysis

**Fig. 2:**
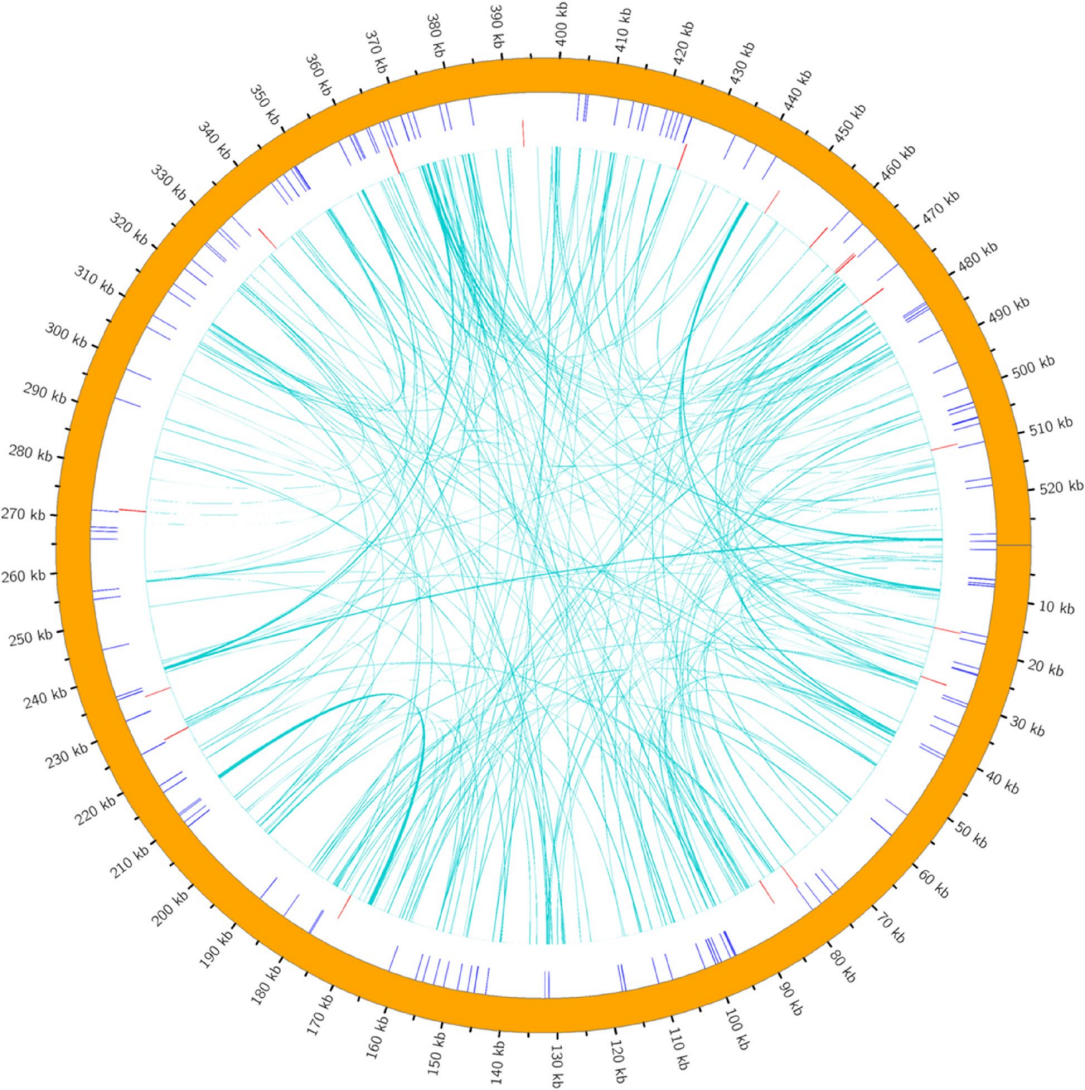
Distribution of repetitive sequences in the *I. metabaptista* mitogenome. The outermost circle is the SSRs, followed by the tandem repeat sequence, and the innermost concatenation is the dispersed repeat sequence

**Fig. 3:**
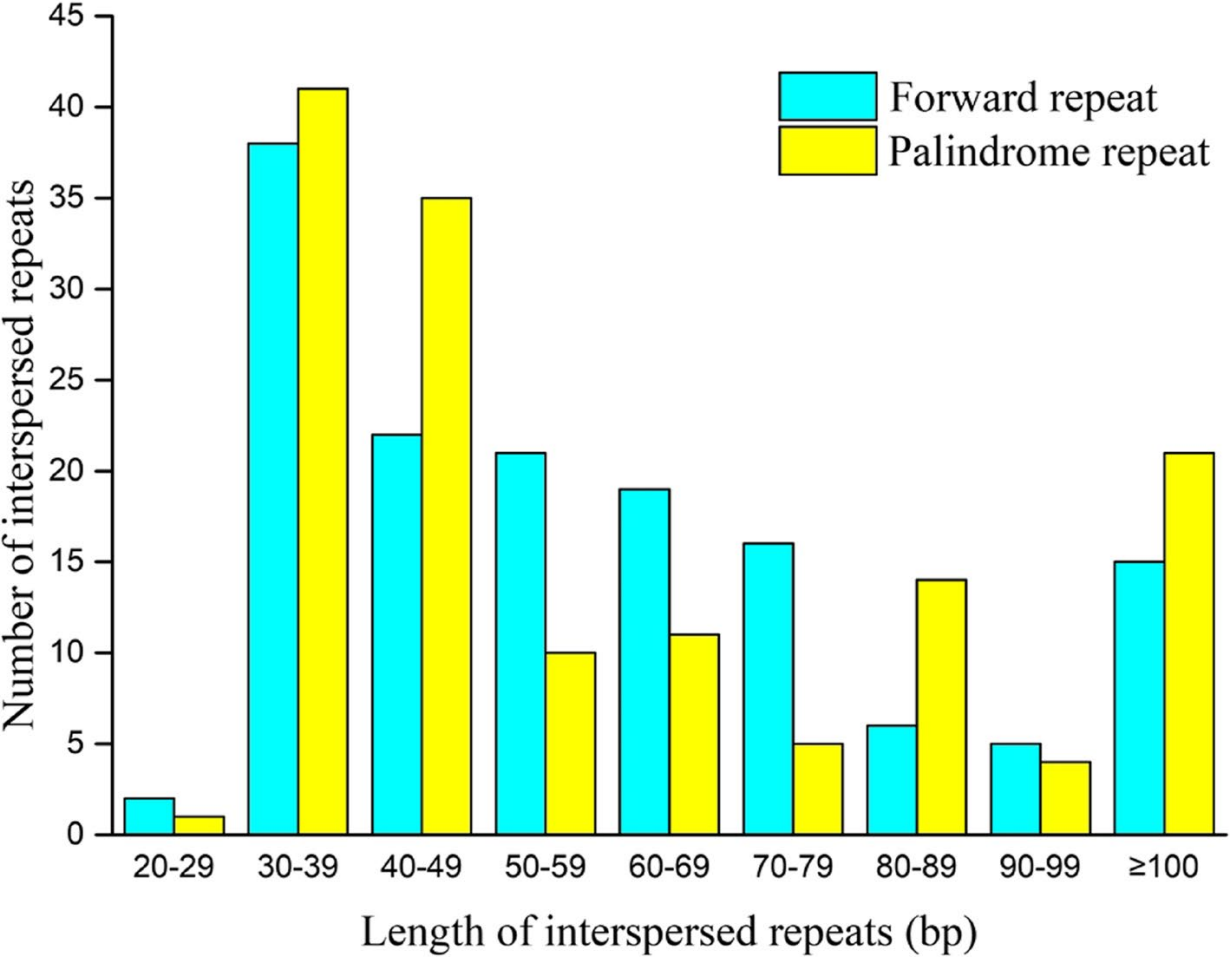
Distribution of lengths of interspersed repeats in the *I. metabaptista* mitogenome

Repeat sequences are abundant in the plant mitogenome, including simple sequence repeats (SSRs), tandem repeats and dispersed repeats [10, 16]. Different types of repeat sequences found in *I. metabaptista* are shown in Fig. 2. Dispersed repeats are repetitive sequences that are scattered throughout the genome [21]. In the *I. metabaptista* mitogenome, a total of 286 dispersed repeats were identified with a length greater than or equal to 29 bp; of these, 144 were forward repeats and 142 were palindromic repeats. The lengths of the longest forward repeat sequence and the longest palindrome repeat sequence were 810 and 413 bp, respectively. The total length of the scattered repetitive sequences was 19,931 bp, accounting for 3.76% of the total length of the mitogenome. The abundance of both types of repeats was the highest when repeats were in the range of 30–39 bp (Fig. 3).

SSRs are DNA fragments with a length of 1–6 bp that are widely used in species research due to their advantages, which include polymorphism, codominant inheritance, relative abundance, and wide genome coverage [16]. As shown in Table 3, we identified 140 SSRs in the *I. metabaptista* mitogenome, and the detected SSR sites included monomer, dimer, trimer, tetramer, and pentamer repeats. Tetramer repeats were the most abundant SSR type, constituting 42.14% of the total identified SSRs, followed by dimer and hexamer repeats, which accounted for 23.57% and 21.43% of the total SSRs, respectively; the number of trimer and pentamer repeats was the lowest. Monomer repeats composed of A/T bases accounted for 93.33% of monomer SSRs, and dimer repeats composed of AG/CT bases accounted for 51.52% of dimer SSRs. There were no hexanucleotide repeats in the *I. metabaptista* mitogenome.

**Table 3:** The SSR types detected in the *I. metabaptista* mitogenome

SSR type	Repeats	Number of SSRs	Total
monomer	A/T	28	30
	C/G	2	
dimer	AG/CT	17	33
	AT/AT	16	
trimer	AAC/GTT	1	11
	AAG/CTT	6	
	ACC/GGT	1	
	AGC/CTG	1	
	AGG/CCT	2	
tetramer	AAAC/GTTT	3	59
	AAAG/CTTT	12	
	AAAT/ATTT	1	
	AAGC/CTTG	7	
	AAGG/CCTT	5	
	AAGT/ACTT	4	
	AATC/ATTG	2	
	AATG/ATTC	7	
	ACAT/ATGT	2	
	ACCG/CGGT	2	
	ACCT/AGGT	1	
	ACTC/AGTG	1	
	ACTG/AGTC	1	
	AGAT/ATCT	2	
	AGCC/CTGG	2	
	AGCG/CGCT	1	
	AGCT/AGCT	4	
	ATCG/ATCG	1	
	CCCG/CGGG	1	
pentamer	AAAAC/GTTTT	1	7
	AAAAG/CTTTT	1	
	AAAGT/ACTTT	1	
	AAGAG/CTCTT	2	
	AAGAT/ATCTT	1	
	ACTAG/AGTCT	1	

Tandem repeats, also known as satellite DNAs, are core repeating units of 1-200 bases repeated several times in tandem and are widely present in eukaryotic and some prokaryotic genomes [34]. As shown in Table 4, a total of 18 tandem repeats ranging in length from 9 to 39 bp that had a match degree greater than 81% were found in the genome.

**Table 4:** Distribution of tandem repeats in the *I. metabaptista* mitogenome

NO.	Size	Repeat sequence	Copy	Percent Matches	Start	End
1	9	2.9	CCTTAAAGG	100	17,464	17,489
2	18	2.4	CATAGTCGCGAGCTGTTT	81	28,112	28,154
3	18	2.3	TTGAACTGATTCGAATCC	82	78,552	78,592
4	15	2.3	TGAAGAGAGGAGGAG	89	84,114	84,147
5	13	2.9	AGAATCATATGAA	88	174,815	174,852
6	23	3.3	GAGGGCTTGTCCTCTCAATCGCC	92	224,868	224,945
7	14	2.1	TTTTATATATCTAG	100	234,173	234,201
8	14	2.1	TGAAAGTATATTAA	100	271,896	271,925
9	15	2.3	AAGTCAAAGCAAGCTCA	86	335,412	335,447
10	20	2	TTTTTTCCTTCTTTATTAAGA	90	365,966	366,006
11	18	1.9	ATTATTACCTAAGGCCTC	100	393,018	393,052
12	39	2	AATATCATGATCGGGTCGACCAGGCCAGATCATGAGTGA	97	425,990	426,068
13	14	2.3	AACTAGGAGAGAAAG	89	446,622	446,654
14	24	2.5	CCTGCTCTACTCCCTACTTTGAGT	94	458,588	458,645
15	18	1.9	TAGGTTTGGTTACAGGAAT	88	465,911	465,946
16	27	2.6	GTACCTACTTGTATTTACCGTAAAAAC	86	466,331	466,400
17	37	2.6	CTTTTGTAGTTGAGGGAACTCGTCCATCCACGGGACAC	93	474,935	475,028
18	13	2.2	TATGTCTGTCAAA	100	509,255	509,283

### Prediction of RNA editing sites

**Fig. 4:**
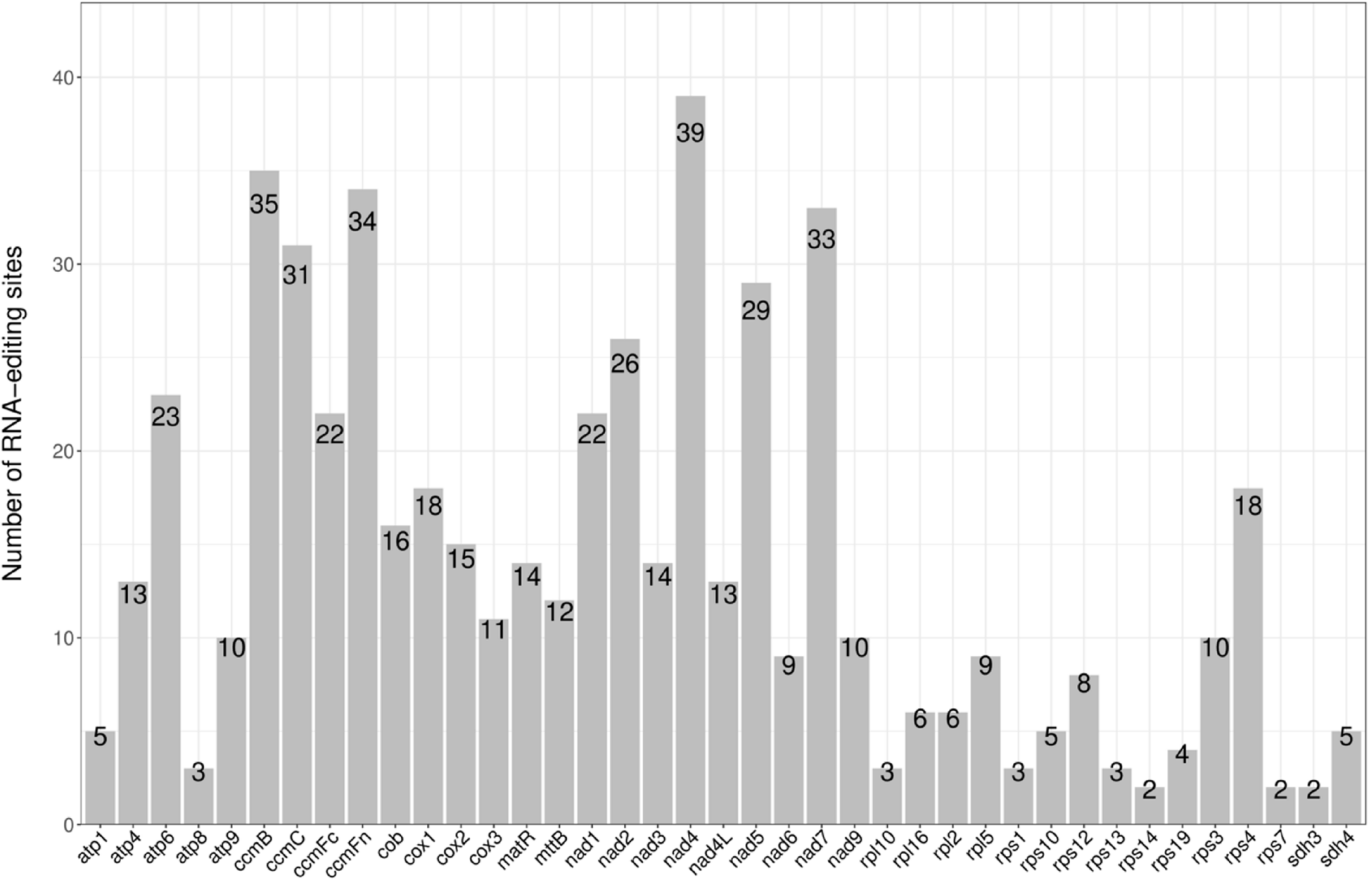
Distribution of RNA editing sites in protein-coding genes of the *I. metabaptista* mitogenome

In all eukaryotes, the addition, loss, or substitution of bases in the coding region of the transcribed RNA is called RNA editing [28]. In this study, a total of 543 RNA editing sites were predicted within 39 PCGs of the *I. metabaptista* mitogenome (Table 5). All RNA editing sites were unevenly distributed among different genes, ranging from 2 (*rps14*, *rps7*, and *sdh3*) to 39 (*nad4*) (Fig. 4). After RNA editing, 43.09% of amino acids were predicted to remain unchanged in hydrophobicity, 8.47% to change from hydrophobic to hydrophilic, and 47.51% to change from hydrophilic to hydrophobic.

There were only 30 codon transfer types, corresponding to 14 amino acid transfer types. Among all codon transfer types, TCA = > TTA was the most common, with 84 sites. The predicted results also showed that the amino acids generated after codon editing had the highest tendency to convert to leucine after RNA editing; 46.22% (251 sites) of amino acids were converted to leucine. All RNA-editing sites in the *I. metabaptista* mitogenome were the C-T editing type; among these, 30.57% (166) of the editing sites were located on the first base of the triplet codon, and 65.75% (357) of the editing sites were located on the second base of the triplet codon. There were two particular editing cases in which both the first and second bases of the triplet codon were edited, resulting in the conversion of proline (CCC, CCT) to phenylalanine (TTC, TTT). However, no editing occurred at the third position of the triplet codons. In addition, 0.92% of the amino acids were edited into a stop codon (TAG, TGA).

**Table 5:** Prediction of RNA editing sites

Type	RNA-editing	Number	Percentage
hydrophilic-hydrophilic	CAC (H) = > TAC (Y)	8	
	CAT (H) = > TAT (Y)	16	
	CGC (R) = > TGC (C)	12	
	CGT (R) = > TGT (C)	29	
	total	65	11.97%
hydrophilic-hydrophobic	ACA (T) = > ATA (I)	3	
	ACC (T) = > ATC (I)	1	
	ACG (T) = > ATG (M)	7	
	ACT (T) = > ATT (I)	4	
	CGG (R) = > TGG (W)	32	
	TCA (S) = > TTA (L)	84	
	TCC (S) = > TTC (F)	33	
	TCG (S) = > TTG (L)	45	
	TCT (S) = > TTT (F)	49	
	total	258	47.51%
hydrophilic-stop	CAG (Q) = > TAG (X)	1	
	CGA (R) = > TGA (X)	4	
	total	5	0.92%
hydrophobic-hydrophilic	CCA (P) = > TCA (S)	10	
	CCC (P) = > TCC (S)	13	
	CCG (P) = > TCG (S)	3	
	CCT (P) = > TCT (S)	20	
	total	46	8.47%
hydrophobic-hydrophobic	CCA (P) = > CTA (L)	52	
	CCC (P) = > CTC (L)	7	
	CCC (P) = > TTC (F)	8	
	CCG (P) = > CTG (L)	37	
	CCT (P) = > CTT (L)	26	
	CCT (P) = > TTT (F)	12	
	CTC (L) = > TTC (F)	7	
	CTT (L) = > TTT (F)	11	
	GCC (A) = > GTC (V)	1	
	GCG (A) = > GTG (V)	5	
	GCT (A) = > GTT (V)	3	
	total	169	31.12%
	All	543	100%

### Analysis of codon usage

We analysed the codon composition of the *I. metabaptista* mitogenome (Table 6). The number of codons in all coding genes was 11,041, and the GC1, GC2, and GC3 content and the average GC content of 3 bases (all GC) were less than 50%, indicating that the codons of the *I. metabaptista* mitogenome were biased because of the use of both A and T bases. The effective codon number (Nc) was 53.24, which indicated that the codon preference of the *I. metabaptista* mitogenome was weak [21]. As shown in Table 1, most PCGs used ATG as the start codon, whereas *nad4L* and *rps10* used ACG as the start codon, presumably a consequence of alteration by RNA editing [16], and *rps4* used TTG as the start codon. The utilization rates of the TAA, TGA, and TAG stop codons were 56.41, 33.33, and 10.26%, respectively. The use rate of the TAA stop codon was the highest.

**Fig. 5:**
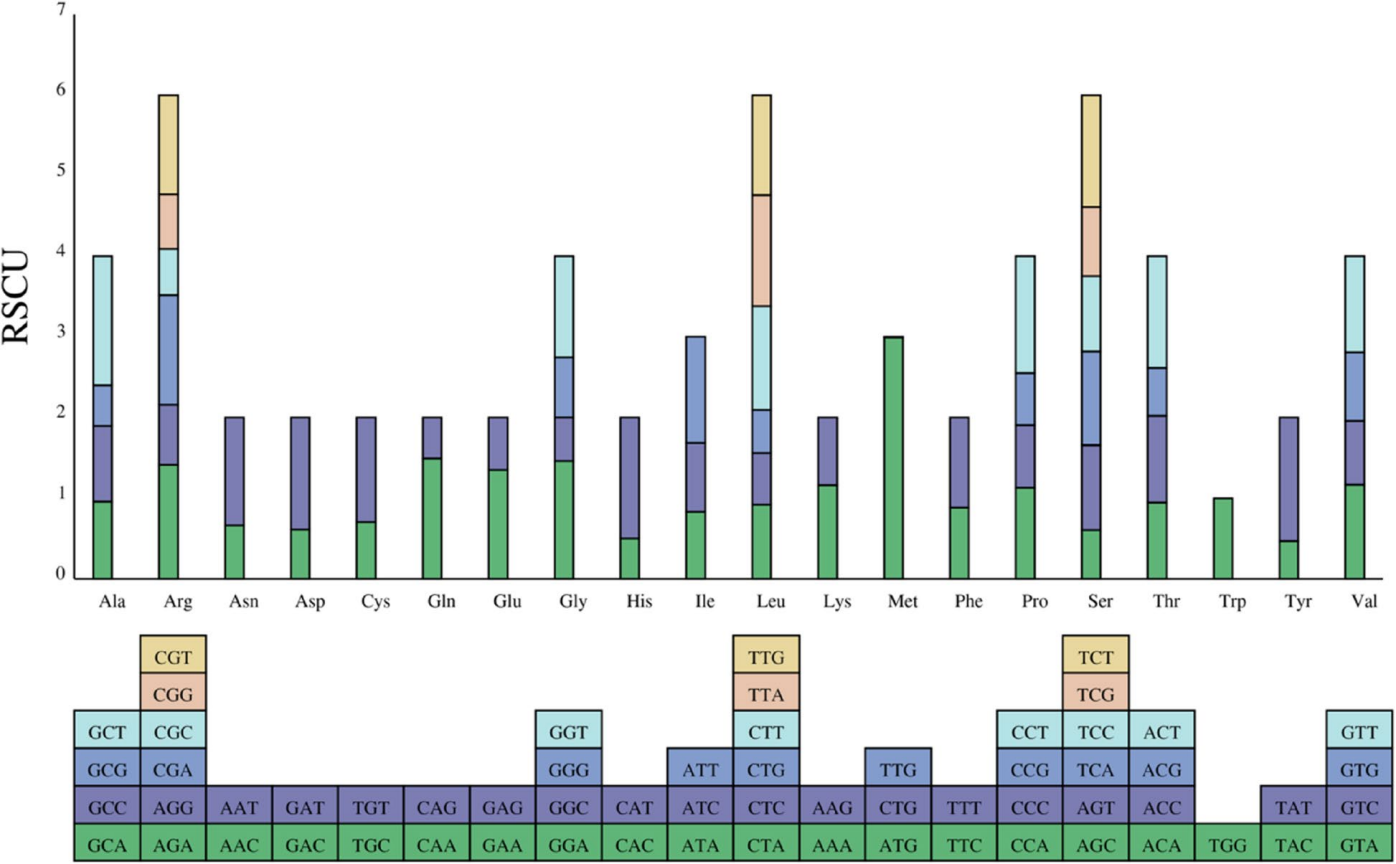
Relative synonymous codon usage (RSCU) in the *I. metabaptista* mitogenome. The different amino acids are shown on the x-axis. RSCU values are the number of times a particular codon is observed relative to the number of times that codon would be expected for uniform synonymous codon usage

The codon usage bias in the *I. metabaptista* mitogenome was measured by calculating the relative synonymous codon usage (RSCU) (Table S2). If RSCU = 1, it indicates that codon usage is unbiased, and if RSCU < 1, it indicates that the actual frequency of use of the codon is lower than the frequency of use of other synonymous codons, and if RSCU > 1, it is higher than the frequency of use of other synonymous codons [21]. As shown in Fig. 5, there were 30 codons with RSCU > 1, indicating that the usage frequency of these codons was greater than that of other synonymous codons. Among these, 27 codons ending with the A/T base were identified, and these accounted for 90.00% of the codons.

**Table 6:** Overall characteristics of codon usage in the *I. metabaptista* mitogenome

Parameter	Codon number	GC1	GC2	GC3	GC_all	Nc
value	11,041	47.94	43.47	38.15	43.18	53.24

### Analysis of homologous fragments between mitochondria and chloroplasts

**Fig. 6:**
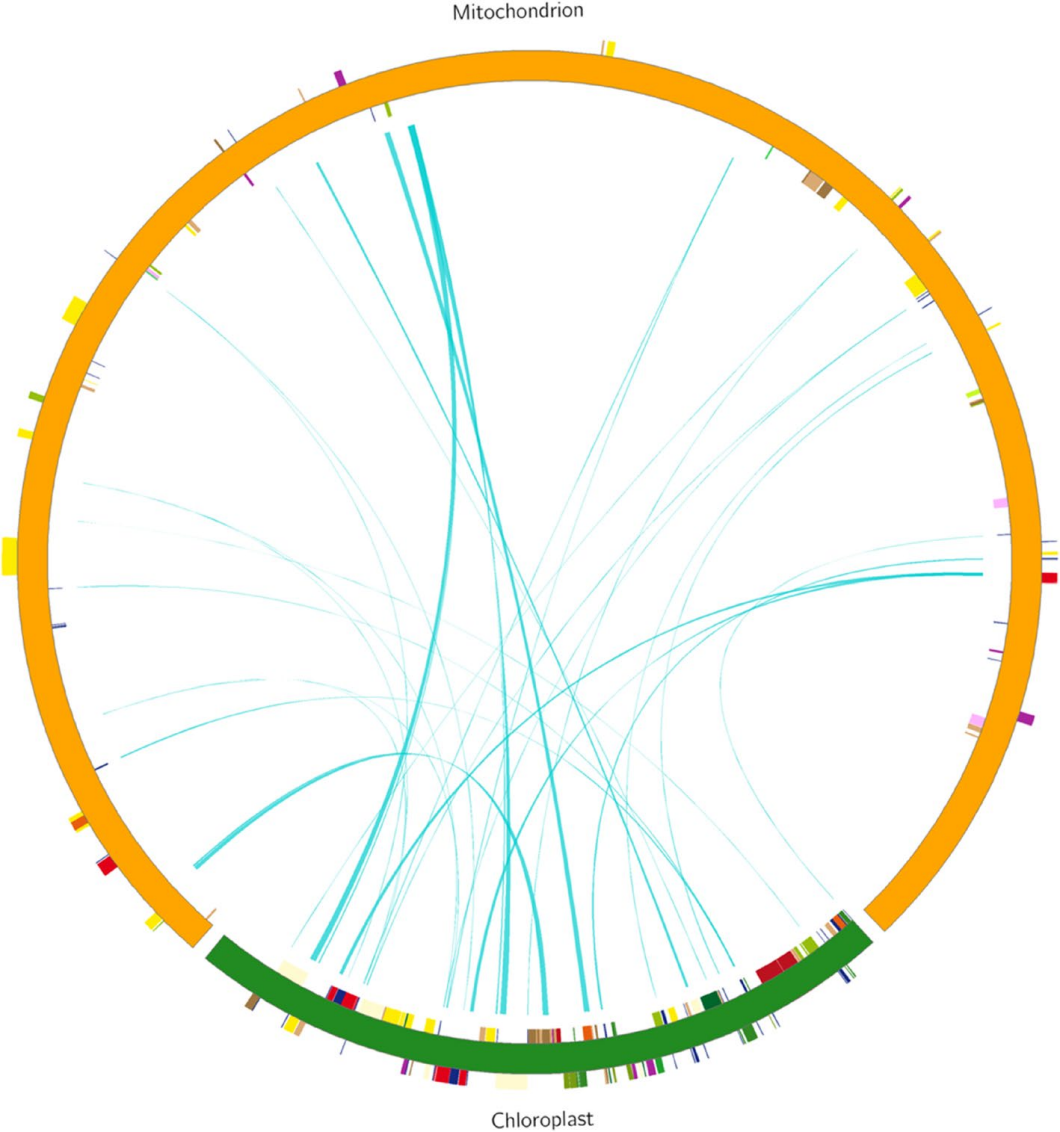
Distribution of homologous fragments between mitochondria and chloroplasts in *I. metabaptista*. The green arcs of the circle represent the chloroplast genome, and the yellow arcs represent the mitogenome. The blue lines between the arcs correspond to the genomic fragments that are homologous

The homologous fragments between the *I. metabaptista* mitogenome and chloroplast genome were detected and analysed (Fig. 6). We screened 30 homologous fragments, ranging in length from 41 to 1,564 bp, with a total length of 11,100 bp, which accounted for 2.10% of the mitogenome (Table 7). One intact chloroplast PCG (*ycf15*), seven tRNA genes (*trnD-GUC*, *trnH-GUG*, *trnI-CAU*, *trnM-CAU*, *trnN-GUU*, *trnW-CCA*, and *trnP-UGG*), and numerous partial genes and intergenic spacer regions were identified.

**Table 7:** Homologous fragments between mitochondria and chloroplasts in *I. metabaptista*

	Alignment Length (bp)	Identity(%)	Mismatch	Gap opens	CP Start	CP End	Mt Start	Mt End	Gene
1	1,564	99.872	1	1	148,408	149,970	261,824	260,261	*ycf15;ycf2*(partical:17.30%)
2	1,564	99.872	1	1	95,131	96,693	260,261	261,824	*ycf2*(partical:17.30%);ycf15
3	1,373	99.199	10	1	72,872	74,243	255,139	253,767	*clpP*(partical:60.11%)
4	971	87.333	91	17	83,831	84,779	13,039	13,999	*rpl14*(partical:87.80%);rpl16(partical:36.89%)
5	431	87.471	34	12	69,297	69,718	482,588	482,169	*trnW-CCA;trnP-UGG*
6	229	99.563	1	0	147,291	147,519	262,042	261,814	*ndhB*(partical:6.60%)
7	229	99.563	1	0	97,582	97,810	261,814	262,042	*ndhB*(partical:6.60%)
8	434	83.641	49	12	84,867	85,286	14,156	14,581	*rpl16*(partical:31.37%)
9	637	78.964	71	34	45,543	46,139	234,415	233,802	*ycf3*(partical:30.11%)
10	375	84.8	29	12	31,950	32,311	49,146	49,505	*trnD-GUC*;t*rnY-GUA*(partical:76.19%)
11	888	73.986	178	39	140,348	141,211	486,879	486,021	*rrn16*(partical:57.99%)
12	888	73.986	178	39	103,890	104,753	486,021	486,879	r*rn16*(partical:57.99%)
13	146	99.315	0	1	134,169	134,313	349,583	349,438	*rrn5*(partical:80.17%)
14	146	99.315	0	1	110,788	110,932	349,438	349,583	*rrn5*(partical:80.17%)
15	147	98.639	2	0	36,659	36,805	425,027	425,173	*psbC*(partical:10.34%)
16	189	89.947	17	1	31,954	32,140	96,500	96,688	*trnD-GUC*
17	93	94.624	4	1	133,139	133,231	411,706	411,615	*trnN-GUU*
18	93	94.624	4	1	111,870	111,962	411,615	411,706	*trnN-GUU*
19	83	95.181	4	0	6	88	476,174	476,256	*trnH-GUG*
20	79	92.405	6	0	54,581	54,659	422,173	422,251	*trnM-CAU*
21	62	93.548	4	0	133,950	134,011	61,918	61,857	
22	62	93.548	4	0	111,090	111,151	61,857	61,918	
23	44	100	0	0	111,882	111,925	124,519	124,476	*trnN-GUU*(partical:56.00%)
24	44	100	0	0	133,176	133,219	124,476	124,519	*trnN-GUU*(partical:56.00%)
25	52	94.231	2	1	11,740	11,791	114,208	114,158	*atpA*(partical:3.41%)
26	45	97.778	1	0	40,394	40,438	221,469	221,425	*psaB*(partical:2.04%)
27	75	86.667	4	1	89,267	89,341	391,020	390,952	*trnI-CAU*
28	75	86.667	4	1	155,760	155,834	390,952	391,020	*trnI-CAU*
29	41	97.561	1	0	138,567	138,607	180,924	180,884	
30	41	97.561	1	0	106,494	106,534	180,884	180,924	
Total	11,100								

### Phylogenetic analysis

**Fig. 7:**
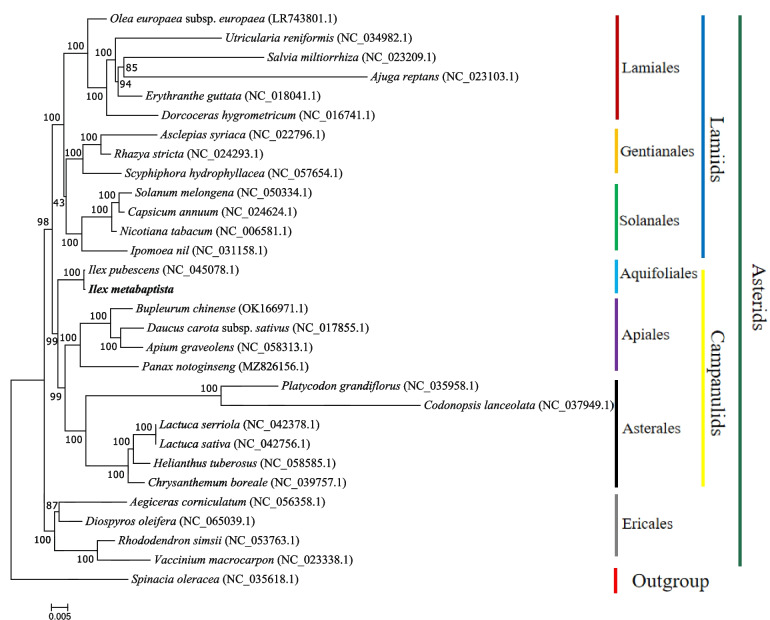
The phylogenetic relationships of *I. metabaptista* with 29 other plant species. *Spinacia oleracea* served as an outgroup. The bootstrap values are listed in each node. The number after the species name is the GenBank accession number. Colours indicate the groups to which the specific species belong

To understand the evolutionary status of the *I. metabaptista* mitogenome, phylogenetic analysis was performed on the *I. metabaptista* mitogenome together with the published mitogenomes of 29 other plants, including 28 asterids and *Spinacia oleracea* (designated as the outgroup). A phylogenetic tree was obtained based on these species, as shown in Fig. 7. As an outgroup, *S. oleracea* was distinct from the asterids. All 7 taxa of the studied orders (Ericales, Gentianales, Solanales, Lamiales, Aquifoliales, Asterales and Apiales) were well clustered. Moreover, the phylogenetic tree strongly supported the separation of campanulids from lamiids and the separation of the basal groups from campanulids and lamiids. In addition, the target tree species *I. metabaptista* and *I. pubescens*, which both belong to the genus *Ilex* in the Aquifoliaceae family, were clustered into a narrow branch with a high bootstrap support value (100%) and formed a sister cluster with the clade of Asterales and Apiales with a high bootstrap support value of 99% (Fig. 7). Consistent with the APG IV taxonomic tree [35], this study also found that Aquifoliales was placed at the base of the campanulids. In general, the clustering in the phylogenetic tree is consistent with the relationships of these species at the order level, indicating that the mitogenome-based clustering results are reliable. Based on the phylogenetic relationships among the 30 species, different groups of plants were selected for further comparative analysis.

### Substitution rates of protein-coding genes

**Fig. 8:**
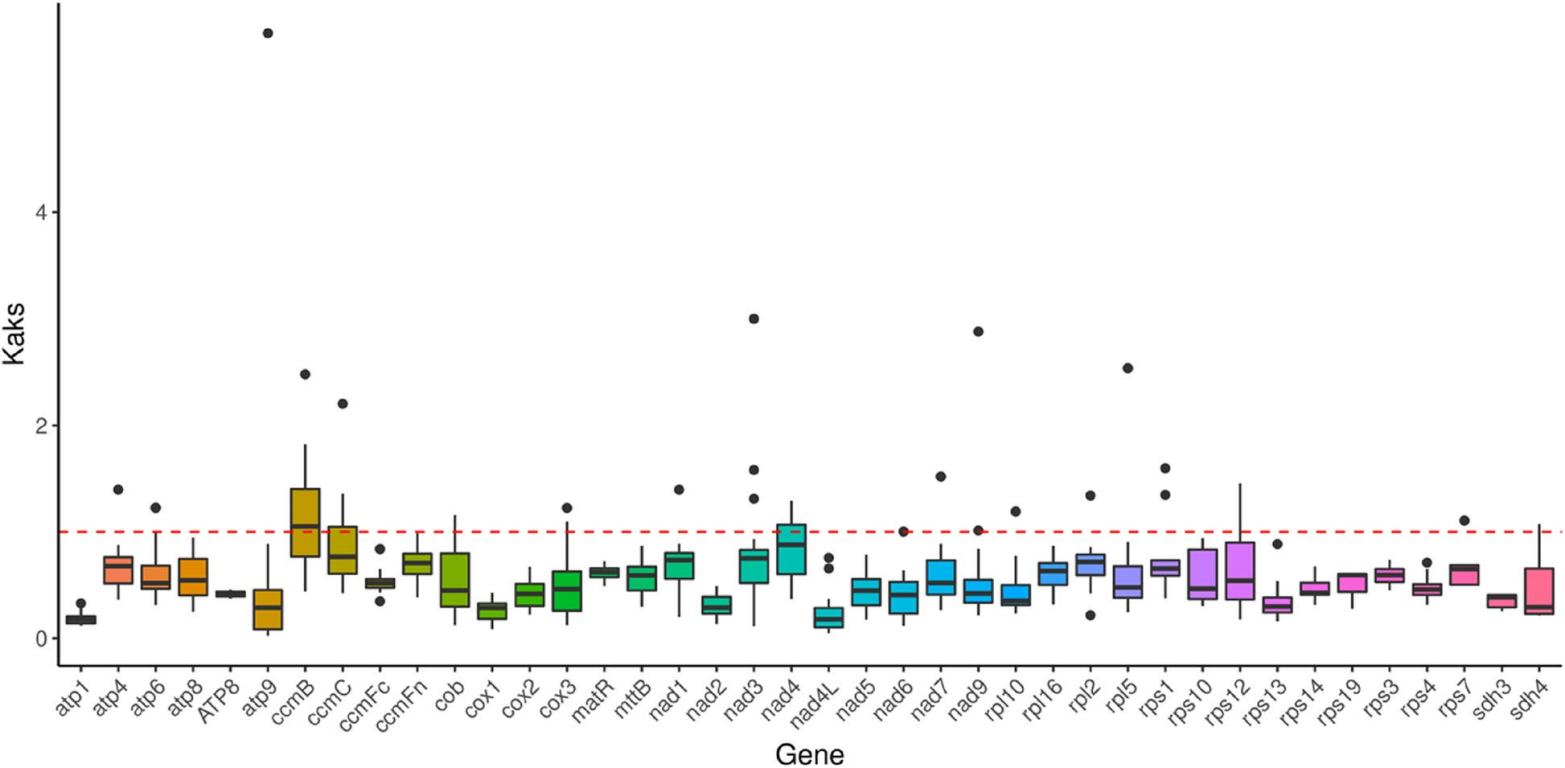
Boxplots of the pairwise Ka/Ks values among every shared mitochondrial gene of the 8 asterids

To evaluate selective pressures during the evolutionary dynamics of PCGs among closely related species, the nonsynonymous (Ka) and synonymous (Ks) substitution ratios (Ka/Ks) were calculated. In the case of neutral selection, Ks = Ka or Ka/Ks = 1. If the Ka value is higher than the Ks value, it is indicative of positive selection (Ka/Ks > 1), while if Ks > Ka or Ka/Ks < 1, it is indicative of negative selection [36, 37]. The 39 PCGs from the *I. metabaptista* mitogenome were compared with the mitogenomes of 7 other asterids for Ka/Ks calculation. As shown in Fig. 8, for the gene-specific substitution rates, Ka/Ks ranged from 0.024 at the *atp9* gene to 5.684 at the *atp9* gene. The *ccmB* gene exhibited the highest average Ka/Ks value (1.112), which was higher than 1, suggesting that positive selection occurred during evolution. However, the Ka/Ks values of most genes were less than 1 in most species, suggesting that they had undergone negative selection during evolution. The *atp1* gene had the smallest average Ka/Ks value (0.185), less than 1.0 in all species, indicating strong purifying selection and high conservation during the evolutionary process in asterids plants [38].

### Nucleotide diversity

**Fig. 9:**
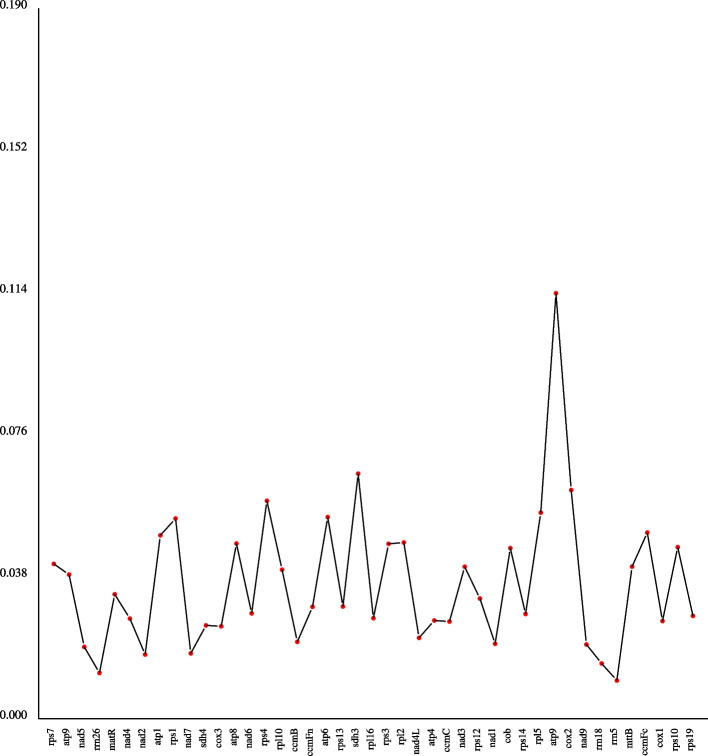
Nucleotide diversity (Pi) among asterid mitogenomes

Nucleotide diversity (Pi) can be used to evaluate the variation in nucleic acid sequences of different species, and regions with higher variability can be selected as potential molecular markers for population genetics [39]. The nucleotide diversity of the 39 PCGs and 3 rRNA genes among the eight asterids is shown in Fig. 9. The Pi values of 42 genes ranged from 0.026 to 0.114, and most of the Pi values were lower than 0.1. Among the PCGs, *atp9* (Pi = 0.114) displayed the highest variability, and *sdh3* (Pi = 0.066) and *cox2* (Pi = 0.061) were also highly variable. In contrast, the most conserved PCGs were *nad2* (Pi = 0.017) and *nad7* (Pi = 0.017). Moreover, three rRNA genes were all conserved, with values of 0.0102 in *rrn5*, 0.012 in *rrn26* and 0.015 in *rrn18*. Overall, the nucleotide diversity of the PCGs was highly variable among the eight asterids.

### Comparison of mitogenome sizes and GC contents with those of other asterids

**Fig. 10:**
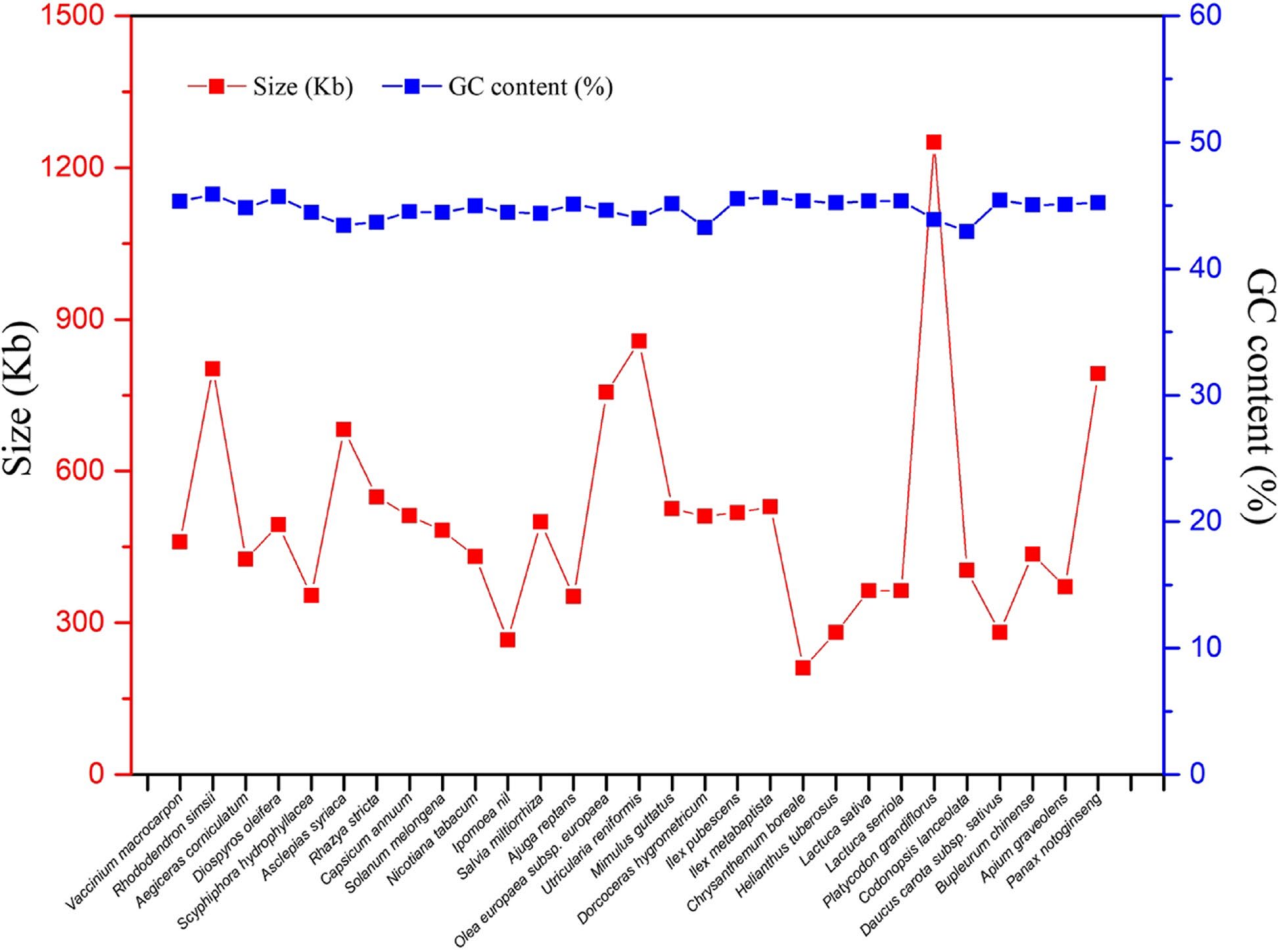
Sizes and GC contents of 29 asterid mitogenomes

The size and GC content of the *I. metabaptista* mitogenome were compared with those of 28 other published asterid mitogenomes (Table S3). As shown in Fig. 10, the genome sizes of the selected asterids varied greatly, ranging from 211,002 bp (*Chrysanthemum boreale*) to 1,249,593 bp (*Platycodon grandiflorus*). The *I. metabaptista* mitogenome was similar to *I. pubescens* in size, which was moderate in size relative to most genomes of asterids (Fig. 10). However, the difference in the GC contents of mitogenomes was relatively small, approximately 45%.

### Comparison of the genome structure with other asterid mitogenomes

**Fig. 11:**
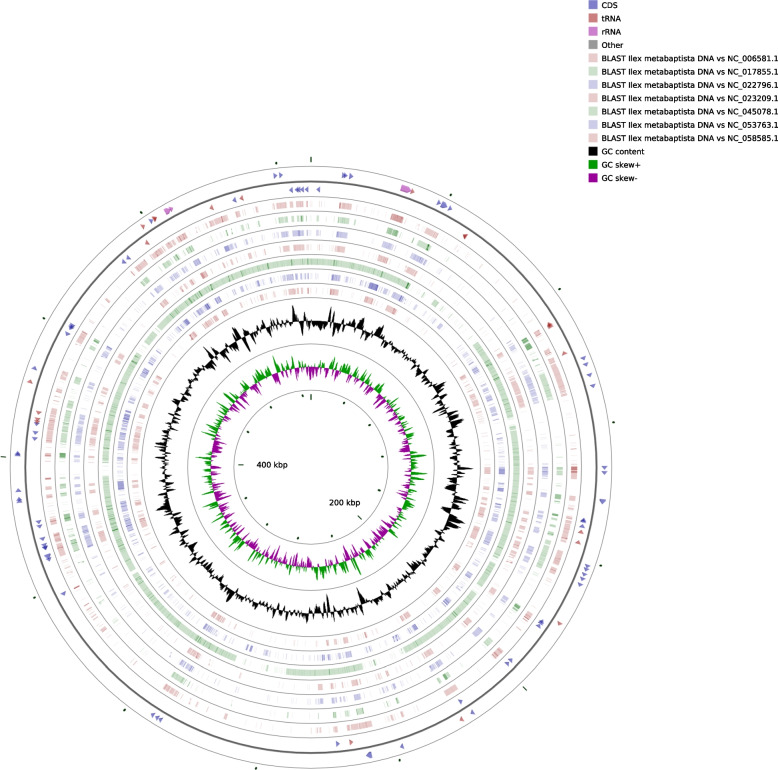
Comparison of *I. metabaptista* mitochondrial structures relative to asterids. The two outermost circles depict the gene length and orientation of the genome; the inner circles represent the similarity results with other reference genomes; the black circles represent the GC content

Because only one mitogenome of a species in Aquifoliaceae has been reported, the mitogenome of *I. metabaptista* was only compared with seven asterids, including one Ericales, one Gentianales, one Solanales, one Lamiales, one Aquifoliales, one Asterales and one Apiales, to further investigate the genome structural variations. As shown in Fig. 11, closely related species shared the most sequences, even outside of the coding regions; species belonging to different groups shared fewer sequences.

### Synteny analysis

**Fig. 12:**
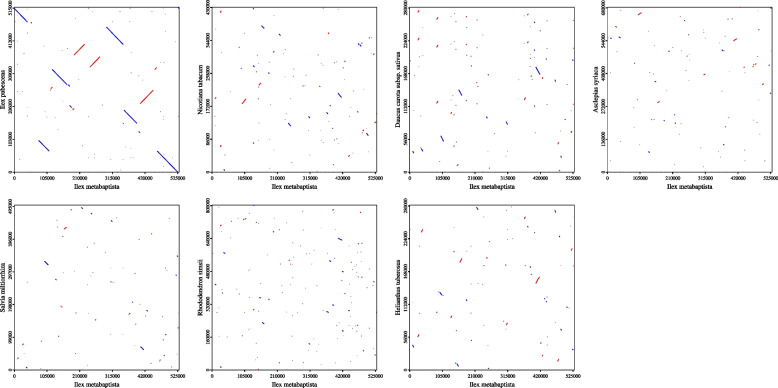
Dot-plot graphs indicating synteny sequences between mitogenomes in asterids compared to *I. metabaptista* as the reference

**Fig. 13:**
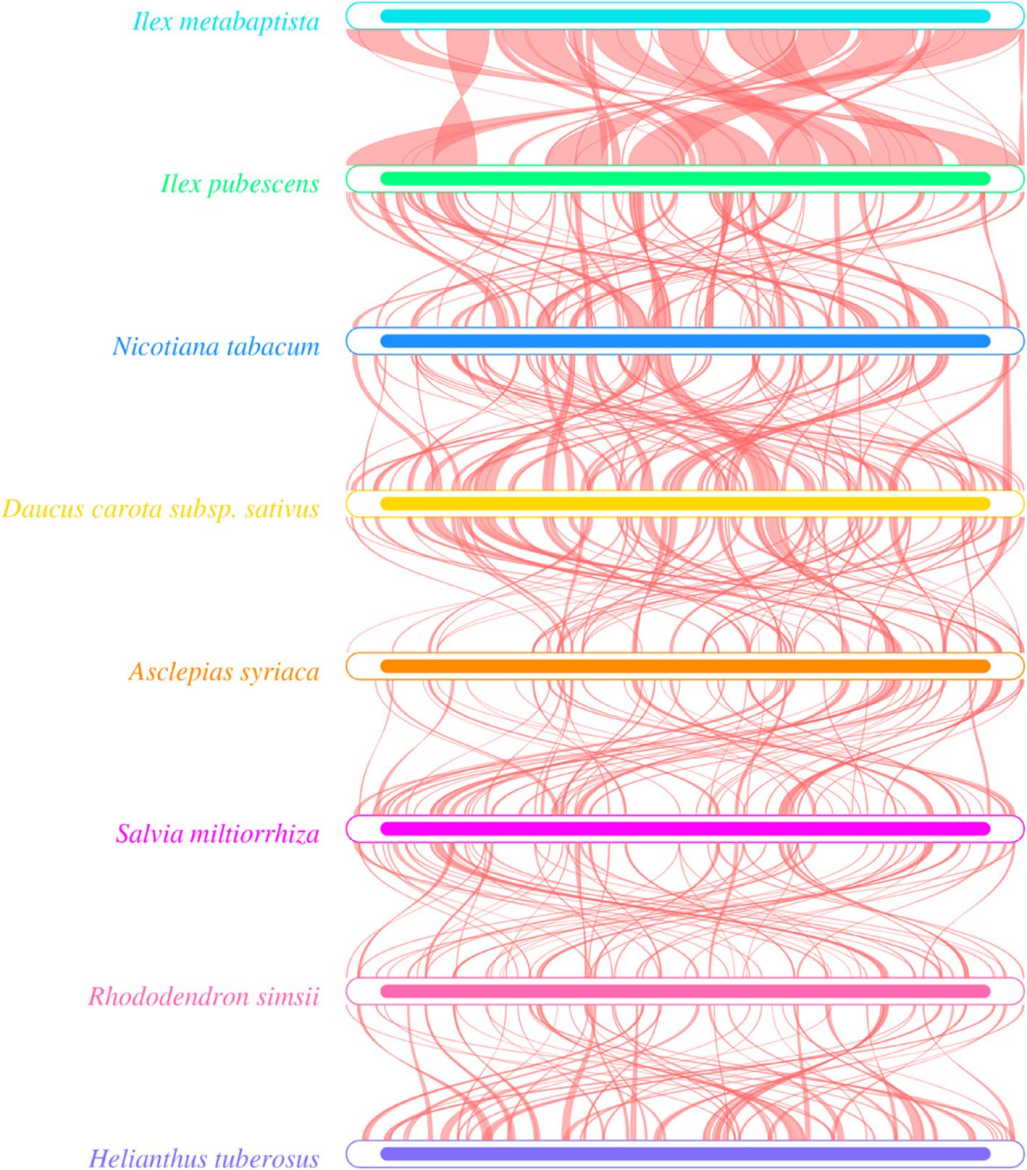
Collinearity plots of *I. metabaptista* and the other seven asterid mitogenomes. The boxes in each row indicate the mitogenomes, and the connecting lines in the middle indicate homologous regions

As shown in Fig. 12, the dot-plot analysis showed that longer synteny sequences with higher similarity were found between *I. metabaptista* and *I. pubescens* than between *I. metabaptista* and other asterids. Pairwise synteny analysis (Fig. 13) showed that there were a large number of homologous colinear blocks, which were not arranged in the same order among individual mitogenomes. These large rearrangement events indicated that the mitogenomes are extremely nonconserved in structure among these eight asterids. Homologous sequences were distributed along the plant mitogenomes, and closely related species shared the most homologous sequences.

## Discussion

### Characterization of the *I*. *metabaptista* mitogenome

Mitochondria provide plant cells with the energy needed for life processes [15]. Plant mitogenomes are fascinating molecules whose variations in noncoding regions and low conservation across species have generated major interest [40]. However, sequencing and analysis of plant mitogenomes are more difficult due to a relatively complex genome characterized by the accumulation of repetitive sequences, incorporation of chloroplast DNA, and extensive rearrangements, which hinder genome assembly [15, 18]. With the rapid development of high-throughput sequencing and assembly technologies, there has been rapid growth in plant mitogenome projects and high-quality mitogenome assemblies in the past several years [16]. The key features of the *I. metabaptista* mitogenome are described in this article. Because of the high recombination frequency, plant mitogenomes have a dynamic structure with various configurations, such as major loops, sub loops and linear molecules, in mitochondria [8, 11]. The *I. metabaptista* mitogenome reported in this study had the typical circular structure of land plant genomes with a length of 529,560 bp and GC content of 45.61%, which were similar to those of *I. pubescens* (517,520 bp; 45.55%) [24].

Repeats are important sources of information for developing markers for population and evolutionary analyses, which are widely present in mitogenomes [16]. Repeats in mitochondrial DNA are generally vital for intermolecular recombination, which plays a crucial role in shaping the mitogenome [33, 41]. Numerous repetitive sequences have been discovered in the mitogenome of *I. metabaptista*, which might indicate the frequent intermolecular recombination frequently occurring in the mitogenome that could dynamically alter the structure and conformation of the mitogenome during evolution [28]. The identified monomer SSRs were mainly composed of the A and T bases connected via two hydrogen bonds, which required less energy to break the bonds than that for the GC bonds [21].

RNA editing occurs during a posttranscriptional process in the mitogenome and chloroplast genome of higher plants and can alter genetic information at the mRNA level [8, 16]. The study of RNA editing sites aids in the comprehension of plant mitochondrial gene expression [33]. In this study, the number of RNA editing sites (543 sites) predicted in the *I. metabaptista* mitogenome was similar to those of other angiosperm plants, such as *Photinia serratifolia* (488) [12], *Diospyros oleifera* (515) [22], and *Sapindus mukorossi* (487) [9], but less than those of gymnosperms, such as *Taxus cuspidata* (974) [42]. However, there were fewer types of codon amino transfer and acid transfer (30 codons; 14 amino acids) than those of angiosperm plants (50–60; approximately 30) [22]. Therefore, the *I. metabaptista* mitogenome has more RNA-editing sites but fewer editing types. Consistent with previous studies, the most abundant transfer type in *I. metabaptista* was TCA = > TTA [7, 21], and the selection of editing sites showed a strong bias, with all editing sites being C-T editing, which is the most common editing type in plant mitogenomes [22]. Additionally, the second position base of the triplet codon was most prone to RNA editing events, and a leucine tendency after RNA editing was found in the amino acids of predicted editing codons [28]. In addition, RNA editing could lead to the premature termination of the coding process in the *I. metabaptista* mitogenome, thus altering the function of the gene [21].

### Mitogenome comparison in asterids

With the rapid development of sequencing technology, an increasing number of complete plant mitogenomes have been assembled and reported recently, facilitating the comparative analysis of mitogenome features among multiple plant species [16, 21]. We compared the genome of *I. metabaptista* to those of other asterids to learn more about its structure and organization. The mitogenomes have undergone extensive rearrangements and are extremely nonconserved in structure among asterids, which might be the main reason for the evolution and diversification of plant mitogenomes [27].

The Ka/Ks analysis and the comparison of genomic features with other plant mitogenomes should contribute to a comprehensive understanding of plant mitochondrial evolution [17]. Generally, consistent with previous studies [21, 22, 28], most of the PCGs in *I. metabaptista* had negative selection during the evolution process, indicating that the PCGs in the mitogenome were relatively well conserved. However, the *ccmB* gene was the only gene that underwent positive selection during evolution, which was consistent with that of *Suaeda glauca* [28]. Other plant mitogenomes also have PCGs with Ka/Ks ratios > 1, and a high gene Ka/Ks ratio plays an important role in further studies on gene selection and evolution of species [38]. In studies of gene selection and evolution in the Aquifoliaceae family, high Ka/Ks gene ratios are very important [21].

The size and GC content are the primary factors for assessing species [7]. We also compared the size and GC content of the *I. metabaptista* mitogenome with those of other asterids. The genome sizes differ greatly, but their GC contents are relatively consistent among asterids, which supports the conclusion that GC contents are highly conserved during the evolutionary process of higher plants [9, 28]. In conclusion, the mitogenome of *I. metabaptista* shares features that are common among other asterids.

### Patterns of codon use bias

Codons play a vital role during transformation of genetic information [15]. There is a wide variation in the rate of genomic codon usage among different species and organisms, which is thought to be the result of a relative equilibrium that gradually develops within the cell over a long period of evolutionary selection [43]. In *I. metabaptista*, most PCGs were the typical ATG start codon, and the distribution of amino acid compositions was similar to other angiosperms [21, 28]. Codon composition analysis showed that the codon preference of the *I. metabaptista* mitogenome was weak, there were 30 codons for which the RSCU > 1, and most of these ended with A/T bases. The results indicated a strong A or T bias in the third position of the codon in the PCGs of the *I. metabaptista* mitogenome; this is commonly observed in plant mitogenomes [21].

### Intergenomic sequence transfers

The evolution of the mitogenome involves many structural rearrangements and gene transfer events [44]. An important feature of plant mitogenome evolution is the transfer of genes between the mitochondria and the chloroplast genomes [16, 45, 46]. Therefore, tracking intergenomic transfer between organellar genomes is essential for understanding the evolution of plant mitogenomes [11, 47]. During mitochondrial evolution, the length and sequence similarity of the migrated fragments vary among higher plants [48]. In this study, the proportion of the transferred fragments between the mitochondria and the chloroplast genomes in *I. metabaptista* was similar to the previously reported data for *Vitex rotundifolia* (2.36%) [49] and *B. chinense* (2.56%) [21] but lower than *Ipomoea batatas* (7.35%) [29]. In addition, tRNA genes are most commonly transferred from the chloroplast genome to the mitogenome in angiosperms [28, 45]. We found that the intracellular tRNA genes transferred frequently from chloroplasts to mitochondria in *I. metabaptista*, which was similar to the results in *S. glauca* [28] and *Acer truncatum* [16]. These findings indicated that tRNA genes were more conserved than PCGs and rRNA genes during evolution since they might remain functional in the mitogenome [43].

### Phylogenetic inference

Because of its many advantages, including maternal inheritance, rapid evolution, low recombination rates, and many available molecular markers, the mitogenome has become a useful tool for the study of taxonomy, phylogeny, evolution, population genetics, and comparative genomics [27, 29]. *Ilex* L. exhibits notable morphological diversity, and the boundaries of some species have not been clearly defined in this genus due to similar morphological features [50, 51]. Thus, further research is needed to understand the origin and evolutionary relationships of this genus. Recently, several studies have characterized the genus *Ilex* by means of phylogeny and biogeography [1], complete chloroplast genome assembly [51], SSR analysis [52], and nuclear genome assembly [30–32]. Aside from this, the taxonomy of the genus is still not clear, and the mitogenomes can help to understand the evolutionary relationships existing among species of the Aquifoliaceae family and the putative hybrid origin for many species within the genus. In the current study, based on the information obtained from the mitogenome, a phylogenetic analysis of the *I. metabaptista* mitogenome and the published mitogenomes of 29 plant species was performed. The evolutionary relationships among these species were consistent with the topology of the phylogenetic tree, indicating the consistency of traditional and molecular taxonomy, which illustrated the possibility of employing information acquired from mitogenomes in plant phylogenetic studies. In addition, these results will lay the foundation for identifying further evolutionary relationships within Aquifoliaceae. However, due to the lack of adequate representative mitogenomes, more mitogenomes of Aquifoliaceae need to be sequenced to better resolve the phylogeny and evolutionary biology within this large family [22].

## Conclusions

In this study, our study produced the first detailed characterization of a complete mitogenome in *Ilex*. The mitogenome of *I. metabaptista* was sequenced, assembled, and annotated, and the DNA and amino acid sequences of annotated genes were analysed thoroughly. The *I. metabaptista* mitogenome was circular and 529,560 bp in length. In addition, 67 genes, of which 42 PCGs, 22 tRNA genes, and 3 rRNA genes, were annotated in the mitogenome. Then, the repeat sequences, RNA-editing sites, homologous fragments between mitochondria and chloroplasts, patterns of biased codon usage, and selective pressure were analysed. Additionally, Ka/Ks analysis, nucleotide polymorphism analysis, and comparative analysis of genomic features were performed to provide a more comprehensive understanding of mitogenome evolution in asterids. Furthermore, the evolutionary status of *I. metabaptista* was verified by phylogenetic analysis based on the mitogenomes of this species and 29 other taxa. This study provides extensive information regarding the *I. metabaptista* mitogenome, which is helpful for future research on the genetic variation, systematic evolution, and breeding of *I. metabaptista*. Therefore, these results help us lay a solid foundation for the cultivation, exploitation, and utilization of this multifunctional tree species.

## Methods

### Plant materials, mitochondrial DNA isolation and genome sequencing

Fresh young leaves of *I. metabaptista* were collected from a female tree (Figure S1) growing in Enshi County, Enshi City, Hubei Province, China (109°36′56.48″ E, 30°33′40.52″ N) by Peng Zhou and Fei Li, which were identified by Dr. Peng Zhou of Jiangsu Academy of Forestry, Nanjing, China. The voucher specimens were stored in the herbarium of Nanjing Forestry University, voucher No. NF2023078. The collection of *I. metabaptista* was permitted by the local government. The use of plant leaves in this study complied with all local, national or international guidelines and legislation concerning research involving plants. Leaves were quickly frozen in liquid nitrogen and then stored at -80 °C prior to DNA isolation.

Total genomic DNA was extracted using a plant genomic DNA kit (Tiangen Biotech, Beijing, China). The DNA purity was detected with a 1.0% agarose gel. Then, the qualified library was sequenced and assembled by applying second- and third-generation sequencing platforms by Nanjing Genepioneer Technology Co., Ltd. (Nanjing, China).

Sequencing was performed following the protocol for the Illumina NovaSeq 6000 platform and the library protocol for Nanopore PromethION sequencing. To obtain a high-quality *I. metabaptista* mitogenome, we used fastp (v0.20.0, https://github.com/OpenGene/fastp) software to filter the raw data, discard the sequencing junction and primer sequences in the reads, filter out reads with an average quality value of less than Q5, filter out reads for which the number (N) was greater than 5, and obtain high-quality reads. The triple sequenced data were filtered using Filtlong (v0.2.1, https://link.zhihu.com/?target=https%3A//github.com/rrwick/Filtlong) software and counted using Perl scripts.

### Assembly and annotation of the mitogenome

Plant mitochondrial genes are very conserved. Taking advantage of this feature, the third-generation comparison software Minimap2 (v2.1) [53] was used to compare the original third-generation data with the reference gene sequence (plant mitochondrial core gene) and screen the sequence with a length greater than 50 bp as the candidate sequence in the alignment. The sequence with more aligned genes (one sequencing sequence contains multiple core genes) and higher alignment quality (covering more complete core genes) was selected as the seed sequence. Compare the original long-read sequencing data with the seed sequence, the sequences with minimum overlap of 1 kb and at least 70% similarity were added to seed sequence, and iteratively align the original data to the seed sequence, so as to obtain all long-read sequencing data of the mitogenome. Then, the third-generation assembly software canu [54] was used to correct the third-generation data obtained, and Bowtie2 (v2.3.5.1) [55] was used to align the second-generation data to the corrected sequence. The default parameter Unicycler (v0.4.8) was used to compare the above second-generation data and the corrected third-generation data for concatenation. Finally, the ringed *I. metabaptista* mitogenome was obtained, and the average depth of assembled mitogenomes was 325×.

Mitogenome annotation was performed using the following steps: the encoded proteins and rRNAs were compared to published plant mitochondrial sequences using BLAST, and further manual adjustments were made based on closely related species. The tRNA was annotated using tRNAscanSE (http://lowelab.ucsc.edu/tRNAscan-SE/) with default settings. ORFs were annotated using Open Reading Frame Finder (http://www.ncbi.nlm.nih.gov/gorf/gorf.html). The circular mitochondrial map was drawn using the Draw Organelle Genome Maps online software (OGDRAW v1.3.1, https://chlorobox.mpimp-golm.mpg.de/OGDraw.html).

### Analysis of repeat sequences

Three kinds of repeats (simple sequence, tandem, and dispersed) were detected in the mitogenome. Simple repetitive sequence analysis was performed using MISA online software [56] (https://webblast.ipk-gatersleben.de/misa/). We identified 10, 5, 4, 3, 3, and 3 repeats with 1, 2, 3, 4, 5, and 6 bases, respectively, in this analysis. Tandem repeats with lengths > 6 bp and > 95% matching repeat units were detected using Tandem Repeats Finder v4.09 software [57] (http://tandem.bu.edu/trf/trf.submit.options.html). The parameters were as follows: 2 7 7 80 10 50 2000 -f -d -m. Dispersed repeats were detected using BLASTN (v2.10.1, parameters: -word size 7, evalue 1e^-5^, remove redundancy, remove tandem duplication). Circos v0.69-5 (http://circos.ca/software/download/) was used to visualize these repeats.

### Codon usage analysis

The codon composition of the mitogenome of *I. metabaptista* was analysed using a self-encoded Perl script to screen for a unique CDS and determine the number of codons per gene, GC content (GC1, GC2, and GC3), average GC content of 3 bases (GC all), effective number of codons (Nc, effective number of codons), and RSCU of synonymous codons.

### Homologous fragment analysis

The chloroplast genome sequence of *I. metabaptista* (NC_069021.1) was downloaded from the NCBI Organelle Genome Resources Database. BLAST software on NCBI was used to identify the homologous fragments between the mitogenome and chloroplast genome. Screening criteria were set as the matching rate ≥ 70%, E-value ≤ 1e^-5^, and length ≥ 30 bp. The results were visualized using circos (v0.69-5).

### RNA editing analyses

The editing sites in the mitochondrial RNA of *I. metabaptista* were identified using the mitochondrial gene-encoding proteins of plants as reference proteins. The analysis was conducted using the Plant Predictive RNA Editor (PREP) suite [58] (http://prep.unl.edu/).

### Phylogenetic tree construction

To acquire the phylogenetic position of *I. metabaptista*, 29 plant mitogenomes (Table S4) were downloaded from the NCBI Organelle Genome Resources database (http://www.ncbi.nlm.nih.gov/genome/organelle/). Among these species, not only were the complete mitogenome sequences of these species for analysis available in NCBI, but they were also placed clearly in taxonomy and were widely used. The shared CDSs of 30 species from different families were aligned using MAFFT (v7.427, --auto mode) software [59]. The aligned sequences were connected end-to-end, trimmed with trimAl (v1.4.rev15, parameter: -gt 0.7), and jmodeltest-2.1.10 software was used to predict the model after trimming and determine that the model was of the GTR type. Then, RAxML v8.2.10 [60] (https://cme.h-its.org/exelixis/software.html) software was used to select the GTRGAMMA model, bootstrap = 1000, and build the maximum likelihood evolutionary tree. *Spinacia oleracea* was designated as an outgroup.

### Synonymous and nonsynonymous substitution ratio analysis

Ka/Ks of PCGs were analysed in the mitogenome of *I. metabaptista* using 7 asterids (Table S4) as references. Homologous protein sequences between *I. metabaptista* and other asterids were obtained using BLASTN. Then, the shared PCGs were aligned using mafft (v7.310, https://mafft.cbrc.jp/alignment/software/), and Ka/Ks was calculated using the Ka/Ks Calculator v2.0 [37] (https://sourceforge.net/projects/kakscalculator2/) software MLWL model. The results of the Ka/Ks values were presented using a boxplot drawn using the R package (ggplot2).

### Nucleotide diversity (pi) analysis

Mafft (v7.427, --auto mode) software was used to compare the homologous gene sequences of different species globally, and dnasp5 [61] was used to calculate the Pi value of each gene.

### Comparative analysis of the mitogenome structure

The *I. metabaptista* mitogenome was used as a reference and compared with seven other mitogenomes of the asterids (Table S4) that properly represent their order. The software CGView [62] (http://stothard.afns.ualberta.ca/cgview_server/) default parameters were applied to a comparative analysis of mitogenome structure for the asterids. Genome alignment between other asterid sequences and *I. metabaptista* sequences was performed using nucmer (4.0.0beta2) software [63] with the maxmatch parameter to generate dot-plot plots. BLASTN (2.10.1) software was used to draw collinearity plots, the word size was set to 7, the e-value was set to 1e^-5^, and fragments larger than 300 bp in length were screened and compared.

### Supplementary Information

**Additional file 1: Table S1.** Summary of sequencing statistics.

**Additional file 2: Table S2.** Relative synonymous codon usage values of the *I. metabaptista *mitogenome.

**Additional file 3: Table S3.** Sizes and GC contents of 29 asterid mitogenomes.

**Additional file 4: Table S4.** GenBank accession numbers of mitogenomes for species sampled in this study.

**Additional file 5: Figure S1.** Morphological characteristics of *I. metabaptista*.

## Data Availability

The raw sequencing data for the Illumina and Nanopore platforms and the mitogenome sequences have been deposited in NCBI (https://www.ncbi.nlm.nih.gov/) with accession numbers PRJNA957559, SAMN34257654, SRR24223046, SRR24259136 and OQ928097, respectively.

## Abbreviations

mt: Mitochondria
cp: Chloroplast
ORF: Open reading frame
PCG: Protein-coding gene
rRNA: Ribosomal RNA
tRNA: Transfer RNA
CDS: Coding sequence
ORF: Open reading frame
SSR: Simple sequence repeat
RSCU: Relative synonymous codon usage
Ka/Ks: Nonsynonymous-to-synonymous substitution ratio
