# Supplementary material for: Assembly and comparative analysis of the complete mitochondrial genome of Ilex metabaptista (Aquifoliaceae), a Chinese endemic species with a narrow distribution
Source: BMC Plant Biol. 2023 Aug 14;23:393. doi: 10.1186/s12870-023-04377-7 (PMC10424370; doi:10.1186/s12870-023-04377-7)
Supplement: Supplementary file 1 — Additional file 1: Table S1. Summary of sequencing statistics. [file 12870_2023_4377_MOESM1_ESM.doc]

**Supplementary Table S1** Summary of sequencing statistics.

| **Next-generation sequencing platform** | | | | | **Third-generation sequencing platform** | | | |
| --- | --- | --- | --- | --- | --- | --- | --- | --- |
| **Clean reads** | **Clean bases**  **(bp)** | **Q20 (%)** | **Q30 (%)** | **GC (%)** | **Total pass reads** | **Total pass bases (bp)** | **Mean length (bp)** | **N50 length (bp)** |
| 41,500,878 | 12,450,263,400 | 97.86 | 93.56 | 37.09 | 1,625,318 | 14,406,042,276 | 8,863 | 21,931 |
