# Supplementary material for: Assembly and comparative analysis of the complete mitochondrial genome of Ilex metabaptista (Aquifoliaceae), a Chinese endemic species with a narrow distribution
Source: BMC Plant Biol. 2023 Aug 14;23:393. doi: 10.1186/s12870-023-04377-7 (PMC10424370; doi:10.1186/s12870-023-04377-7)
Supplement: Supplementary file 2 — Additional file 2: Table S2. Relative synonymous codon usage values of the I. metabaptista mitogenome. [file 12870_2023_4377_MOESM2_ESM.doc]

**Supplementary Table S2** Relative synonymous codon usage values of the *I. metabaptista* mitogenome.

| **Amino Acid** | **Symbol** | **Codon** | **No.** | **RSCU** |
| --- | --- | --- | --- | --- |
| * | Ter | UAA | 22 | 1.6923 |
| * | Ter | UAG | 4 | 0.3077 |
| * | Ter | UGA | 13 | 1 |
| A | Ala | GCA | 163 | 0.9602 |
| A | Ala | GCC | 158 | 0.9308 |
| A | Ala | GCG | 86 | 0.5066 |
| A | Ala | GCU | 272 | 1.6024 |
| C | Cys | UGC | 56 | 0.7044 |
| C | Cys | UGU | 103 | 1.2956 |
| D | Asp | GAC | 106 | 0.6127 |
| D | Asp | GAU | 240 | 1.3873 |
| E | Glu | GAA | 310 | 1.3508 |
| E | Glu | GAG | 149 | 0.6492 |
| F | Phe | UUC | 302 | 0.8856 |
| F | Phe | UUU | 380 | 1.1144 |
| G | Gly | GGA | 271 | 1.4629 |
| G | Gly | GGC | 99 | 0.5344 |
| G | Gly | GGG | 138 | 0.7449 |
| G | Gly | GGU | 233 | 1.2578 |
| H | His | CAC | 66 | 0.5 |
| H | His | CAU | 198 | 1.5 |
| I | Ile | AUA | 231 | 0.8339 |
| I | Ile | AUC | 235 | 0.8484 |
| I | Ile | AUU | 365 | 1.3177 |
| K | Lys | AAA | 278 | 1.1632 |
| K | Lys | AAG | 200 | 0.8368 |
| L | Leu | CUA | 168 | 0.9189 |
| L | Leu | CUC | 118 | 0.6454 |
| L | Leu | CUG | 97 | 0.5305 |
| L | Leu | CUU | 235 | 1.2853 |
| L | Leu | UUA | 252 | 1.3783 |
| L | Leu | UUG | 227 | 1.2416 |
| M | Met | AUG | 289 | 2.9897 |
| M | Met | CUG | 0 | 0 |
| M | Met | UUG | 1 | 0.0103 |
| N | Asn | AAC | 113 | 0.6647 |
| N | Asn | AAU | 227 | 1.3353 |
| P | Pro | CCA | 174 | 1.1299 |
| P | Pro | CCC | 119 | 0.7727 |
| P | Pro | CCG | 100 | 0.6494 |
| P | Pro | CCU | 223 | 1.4481 |
| Q | Gln | CAA | 229 | 1.4967 |
| Q | Gln | CAG | 77 | 0.5033 |
| R | Arg | AGA | 178 | 1.4164 |
| R | Arg | AGG | 93 | 0.7401 |
| R | Arg | CGA | 171 | 1.3607 |
| R | Arg | CGC | 72 | 0.5729 |
| R | Arg | CGG | 85 | 0.6764 |
| R | Arg | CGU | 155 | 1.2334 |
| S | Ser | AGC | 101 | 0.6078 |
| S | Ser | AGU | 174 | 1.0471 |
| S | Ser | UCA | 193 | 1.1615 |
| S | Ser | UCC | 156 | 0.9388 |
| S | Ser | UCG | 142 | 0.8546 |
| S | Ser | UCU | 231 | 1.3902 |
| T | Thr | ACA | 130 | 0.9489 |
| T | Thr | ACC | 147 | 1.073 |
| T | Thr | ACG | 81 | 0.5912 |
| T | Thr | ACU | 190 | 1.3869 |
| V | Val | GUA | 194 | 1.1669 |
| V | Val | GUC | 131 | 0.788 |
| V | Val | GUG | 142 | 0.8541 |
| V | Val | GUU | 198 | 1.191 |
| W | Trp | UGG | 157 | 1 |
| Y | Tyr | UAC | 77 | 0.4695 |
| Y | Tyr | UAU | 251 | 1.5305 |
