# Supplementary material for: Assembly and comparative analysis of the complete mitochondrial genome of Ilex metabaptista (Aquifoliaceae), a Chinese endemic species with a narrow distribution
Source: BMC Plant Biol. 2023 Aug 14;23:393. doi: 10.1186/s12870-023-04377-7 (PMC10424370; doi:10.1186/s12870-023-04377-7)
Supplement: Supplementary file 3 — Additional file 3: Table S3. Sizes and GC contents of 29 asterid mitogenomes. [file 12870_2023_4377_MOESM3_ESM.doc]

**Supplementary Table S3** Sizes and GC contents of 29 asterid mitogenomes.

| **Species** | **Accession number** | **Size (bp)** | **GC content (%)** |
| --- | --- | --- | --- |
| *Vaccinium macrocarpon* | NC_023338.1 | 459.678 | 45.33 |
| *Rhododendron simsii* | NC_053763.1 | 802.707 | 45.87 |
| *Aegiceras corniculatum* | NC_056358.1 | 425.282 | 44.82 |
| *Diospyros oleifera* | NC_065039.1 | 493.958 | 45.7 |
| *Scyphiphora hydrophyllacea* | NC_057654.1 | 354.155 | 44.43 |
| *Asclepias syriaca* | NC_022796.1 | 682.498 | 43.43 |
| *Rhazya stricta* | NC_024293.1 | 548.608 | 43.68 |
| *Capsicum annuum* | NC_024624.1 | 511.53 | 44.52 |
| *Solanum melongena* | NC_050334.1 | 482.343 | 44.43 |
| *Nicotiana tabacum* | NC_006581.1 | 430.597 | 44.96 |
| *Ipomoea nil* | NC_031158.1 | 265.768 | 44.45 |
| *Salvia miltiorrhiza* | NC_023209.1 | 499.236 | 44.39 |
| *Ajuga reptans* | NC_023103.1 | 352.069 | 45.10 |
| *Olea europaea* subsp. *europaea* | LR743801.1 | 755.572 | 44.62 |
| *Utricularia reniformis* | NC_034982.1 | 857.234 | 43.98 |
| *Mimulus guttatus* | NC_018041.1 | 525.671 | 45.14 |
| *Dorcoceras hygrometricum* | NC_016741.1 | 510.519 | 43.27 |
| *Ilex pubescens* |  | 517.52 | 45.55 |
| *Ilex metabaptista* | NC_045078.1 | 529.56 | 45.61 |
| *Chrysanthemum boreale* | NC_039757.1 | 211.002 | 45.36 |
| *Helianthus tuberosus* | NC_058585.1 | 281.287 | 45.21 |
| *Lactuca sativa* | NC_042756.1 | 363.324 | 45.35 |
| *Lactuca serriola* | NC_042378.1 | 363.328 | 45.36 |
| *Platycodon grandiflorus* | NC_035958.1 | 1249.593 | 43.89 |
| *Codonopsis lanceolata* | NC_037949.1 | 403.704 | 42.94 |
| *Daucus carota* subsp. *sativus* | NC_017855.1 | 281.132 | 45.42 |
| *Bupleurum chinense* | OK166971.1 | 435.023 | 45.04 |
| *Apium graveolens* | NC_058313.1 | 371.275 | 45.09 |
| *Panax notoginseng* | MZ826156.1 | 792.375 | 45.22 |
