# Supplementary material for: Assembly and comparative analysis of the complete mitochondrial genome of Ilex metabaptista (Aquifoliaceae), a Chinese endemic species with a narrow distribution
Source: BMC Plant Biol. 2023 Aug 14;23:393. doi: 10.1186/s12870-023-04377-7 (PMC10424370; doi:10.1186/s12870-023-04377-7)
Supplement: Supplementary file 4 — Additional file 4: Table S4. GenBank accession numbers of mitogenomes for species sampled in this study. [file 12870_2023_4377_MOESM4_ESM.doc]

**Supplementary Table S4** GenBank accession numbers of mitogenomes for species sampled in this study.

| **Classification** | **Status** |  | **Order** | **Family** | **Species** | **Accession number** |
| --- | --- | --- | --- | --- | --- | --- |
| Ingroup | [Asterids](http://www.iplant.cn/info/Asterids) |  | [Ericales](http://www.iplant.cn/info/Ericales) | Ericaceae | *Vaccinium macrocarpon* | NC_023338.1 |
|  | [Asterids](http://www.iplant.cn/info/Asterids) |  | [Ericales](http://www.iplant.cn/info/Ericales) | Ericaceae | *Rhododendron simsii* # | NC_053763.1 |
|  | [Asterids](http://www.iplant.cn/info/Asterids) |  | [Ericales](http://www.iplant.cn/info/Ericales) | [Primulaceae](http://www.iplant.cn/info/Primulaceae) | *Aegiceras corniculatum* | NC_056358.1 |
|  | [Asterids](http://www.iplant.cn/info/Asterids) |  | [Ericales](http://www.iplant.cn/info/Ericales) | [Ebenaceae](http://www.iplant.cn/info/Ebenaceae) | *Diospyros oleifera* | NC_065039.1 |
|  | [Asterids](http://www.iplant.cn/info/Asterids) | [Lamiids](http://www.iplant.cn/info/Lamiids) | [Gentianales](http://www.iplant.cn/info/Gentianales) | [Rubiaceae](http://www.iplant.cn/info/Rubiaceae) | *Scyphiphora hydrophyllacea* | NC_057654.1 |
|  | [Asterids](http://www.iplant.cn/info/Asterids) | [Lamiids](http://www.iplant.cn/info/Lamiids) | [Gentianales](http://www.iplant.cn/info/Gentianales) | [Apocynaceae](http://www.iplant.cn/info/Apocynaceae) | *Asclepias syriaca* # | NC_022796.1 |
|  | [Asterids](http://www.iplant.cn/info/Asterids) | [Lamiids](http://www.iplant.cn/info/Lamiids) | [Gentianales](http://www.iplant.cn/info/Gentianales) | [Apocynaceae](http://www.iplant.cn/info/Apocynaceae) | *Rhazya stricta* | NC_024293.1 |
|  | [Asterids](http://www.iplant.cn/info/Asterids) | [Lamiids](http://www.iplant.cn/info/Lamiids) | [Solanales](http://www.iplant.cn/info/Solanales) | [Solanaceae](http://www.iplant.cn/info/Solanaceae) | *Capsicum annuum* | NC_024624.1 |
|  | [Asterids](http://www.iplant.cn/info/Asterids) | [Lamiids](http://www.iplant.cn/info/Lamiids) | [Solanales](http://www.iplant.cn/info/Solanales) | [Solanaceae](http://www.iplant.cn/info/Solanaceae) | *Solanum melongena* | NC_050334.1 |
|  | [Asterids](http://www.iplant.cn/info/Asterids) | [Lamiids](http://www.iplant.cn/info/Lamiids) | [Solanales](http://www.iplant.cn/info/Solanales) | [Solanaceae](http://www.iplant.cn/info/Solanaceae) | *Nicotiana tabacum* # | NC_006581.1 |
|  | [Asterids](http://www.iplant.cn/info/Asterids) | [Lamiids](http://www.iplant.cn/info/Lamiids) | [Solanales](http://www.iplant.cn/info/Solanales) | [Convolvulaceae](http://www.iplant.cn/info/Convolvulaceae) | *Ipomoea nil* | NC_031158.1 |
|  | [Asterids](http://www.iplant.cn/info/Asterids) | [Lamiids](http://www.iplant.cn/info/Lamiids) | [Lamiales](http://www.iplant.cn/info/Lamiales) | [Lamiaceae](http://www.iplant.cn/info/Lamiaceae) | *Salvia miltiorrhiza* # | NC_023209.1 |
|  | [Asterids](http://www.iplant.cn/info/Asterids) | [Lamiids](http://www.iplant.cn/info/Lamiids) | [Lamiales](http://www.iplant.cn/info/Lamiales) | [Lamiaceae](http://www.iplant.cn/info/Lamiaceae) | *Ajuga reptans* | NC_023103.1 |
|  | [Asterids](http://www.iplant.cn/info/Asterids) | [Lamiids](http://www.iplant.cn/info/Lamiids) | [Lamiales](http://www.iplant.cn/info/Lamiales) | [Oleaceae](http://www.iplant.cn/info/Oleaceae) | *Olea europaea* subsp. *europaea* | LR743801.1 |
|  | [Asterids](http://www.iplant.cn/info/Asterids) | [Lamiids](http://www.iplant.cn/info/Lamiids) | [Lamiales](http://www.iplant.cn/info/Lamiales) | [Lentibulariaceae](http://www.iplant.cn/info/Lentibulariaceae) | *Utricularia reniformis* | NC_034982.1 |
|  | [Asterids](http://www.iplant.cn/info/Asterids) | [Lamiids](http://www.iplant.cn/info/Lamiids) | [Lamiales](http://www.iplant.cn/info/Lamiales) | [Scrophulariaceae](http://www.iplant.cn/info/Scrophulariaceae) | *Mimulus guttatus* | NC_018041.1 |
|  | [Asterids](http://www.iplant.cn/info/Asterids) | [Lamiids](http://www.iplant.cn/info/Lamiids) | [Lamiales](http://www.iplant.cn/info/Lamiales) | [Gesneriaceae](http://www.iplant.cn/info/Gesneriaceae) | *Dorcoceras hygrometricum* | NC_016741.1 |
|  | [Asterids](http://www.iplant.cn/info/Asterids) | [Campanulids](http://www.iplant.cn/info/Campanulids) | [Aquifoliales](http://www.iplant.cn/info/Aquifoliales) | [Aquifoliaceae](http://www.iplant.cn/info/Aquifoliaceae) | *Ilex metabaptista* ∗# |  |
|  | [Asterids](http://www.iplant.cn/info/Asterids) | [Campanulids](http://www.iplant.cn/info/Campanulids) | [Aquifoliales](http://www.iplant.cn/info/Aquifoliales) | [Aquifoliaceae](http://www.iplant.cn/info/Aquifoliaceae) | *Ilex pubescens* # | NC_045078.1 |
|  | [Asterids](http://www.iplant.cn/info/Asterids) | [Campanulids](http://www.iplant.cn/info/Campanulids) | [Asterales](http://www.iplant.cn/info/Asterales) | [Compositae](http://www.iplant.cn/info/Compositae) | *Chrysanthemum boreale* | NC_039757.1 |
|  | [Asterids](http://www.iplant.cn/info/Asterids) | [Campanulids](http://www.iplant.cn/info/Campanulids) | [Asterales](http://www.iplant.cn/info/Asterales) | [Compositae](http://www.iplant.cn/info/Compositae) | *Helianthus tuberosus* | NC_058585.1 |
|  | [Asterids](http://www.iplant.cn/info/Asterids) | [Campanulids](http://www.iplant.cn/info/Campanulids) | [Asterales](http://www.iplant.cn/info/Asterales) | [Compositae](http://www.iplant.cn/info/Compositae) | *Lactuca sativa* # | NC_042756.1 |
|  | [Asterids](http://www.iplant.cn/info/Asterids) | [Campanulids](http://www.iplant.cn/info/Campanulids) | [Asterales](http://www.iplant.cn/info/Asterales) | [Compositae](http://www.iplant.cn/info/Compositae) | *Lactuca serriola* | NC_042378.1 |
|  | [Asterids](http://www.iplant.cn/info/Asterids) | [Campanulids](http://www.iplant.cn/info/Campanulids) | [Asterales](http://www.iplant.cn/info/Asterales) | [Campanulaceae](http://www.iplant.cn/info/Campanulaceae) | *Platycodon grandiflorus* | NC_035958.1 |
|  | [Asterids](http://www.iplant.cn/info/Asterids) | [Campanulids](http://www.iplant.cn/info/Campanulids) | [Asterales](http://www.iplant.cn/info/Asterales) | [Campanulaceae](http://www.iplant.cn/info/Campanulaceae) | *Codonopsis lanceolata* | NC_037949.1 |
|  | [Asterids](http://www.iplant.cn/info/Asterids) | [Campanulids](http://www.iplant.cn/info/Campanulids) | [Apiales](http://www.iplant.cn/info/Apiales) | [Umbelliferae](http://www.iplant.cn/info/Umbelliferae) | *Daucus carota* subsp. *sativus* # | NC_017855.1 |
|  | [Asterids](http://www.iplant.cn/info/Asterids) | [Campanulids](http://www.iplant.cn/info/Campanulids) | [Apiales](http://www.iplant.cn/info/Apiales) | [Umbelliferae](http://www.iplant.cn/info/Umbelliferae) | *Bupleurum chinense* | OK166971.1 |
|  | [Asterids](http://www.iplant.cn/info/Asterids) | [Campanulids](http://www.iplant.cn/info/Campanulids) | [Apiales](http://www.iplant.cn/info/Apiales) | [Umbelliferae](http://www.iplant.cn/info/Umbelliferae) | *Apium graveolens* | NC_058313.1 |
|  | [Asterids](http://www.iplant.cn/info/Asterids) | [Campanulids](http://www.iplant.cn/info/Campanulids) | [Apiales](http://www.iplant.cn/info/Apiales) | [Araliaceae](http://www.iplant.cn/info/Araliaceae) | *Panax notoginseng* | MZ826156.1 |
| Outgroup |  |  | [Caryophyllales](http://www.iplant.cn/info/Caryophyllales) | [Amaranthaceae](http://www.iplant.cn/info/Amaranthaceae) | *Spinacia oleracea* | NC_035618.1 |

∗Represents the new mitogenome in this study. #Represents these species were used for selective pressure analysis, nucleotide diversity analysis and genomic comparison.
