# Supplementary material for: Assembly and comparative analysis of the complete mitochondrial genome of Ilex metabaptista (Aquifoliaceae), a Chinese endemic species with a narrow distribution
Source: BMC Plant Biol. 2023 Aug 14;23:393. doi: 10.1186/s12870-023-04377-7 (PMC10424370; doi:10.1186/s12870-023-04377-7)
Supplement: Supplementary file 5 — Additional file 5: Figure S1. Morphological characteristics of I. metabaptista. [file 12870_2023_4377_MOESM5_ESM.doc]

| 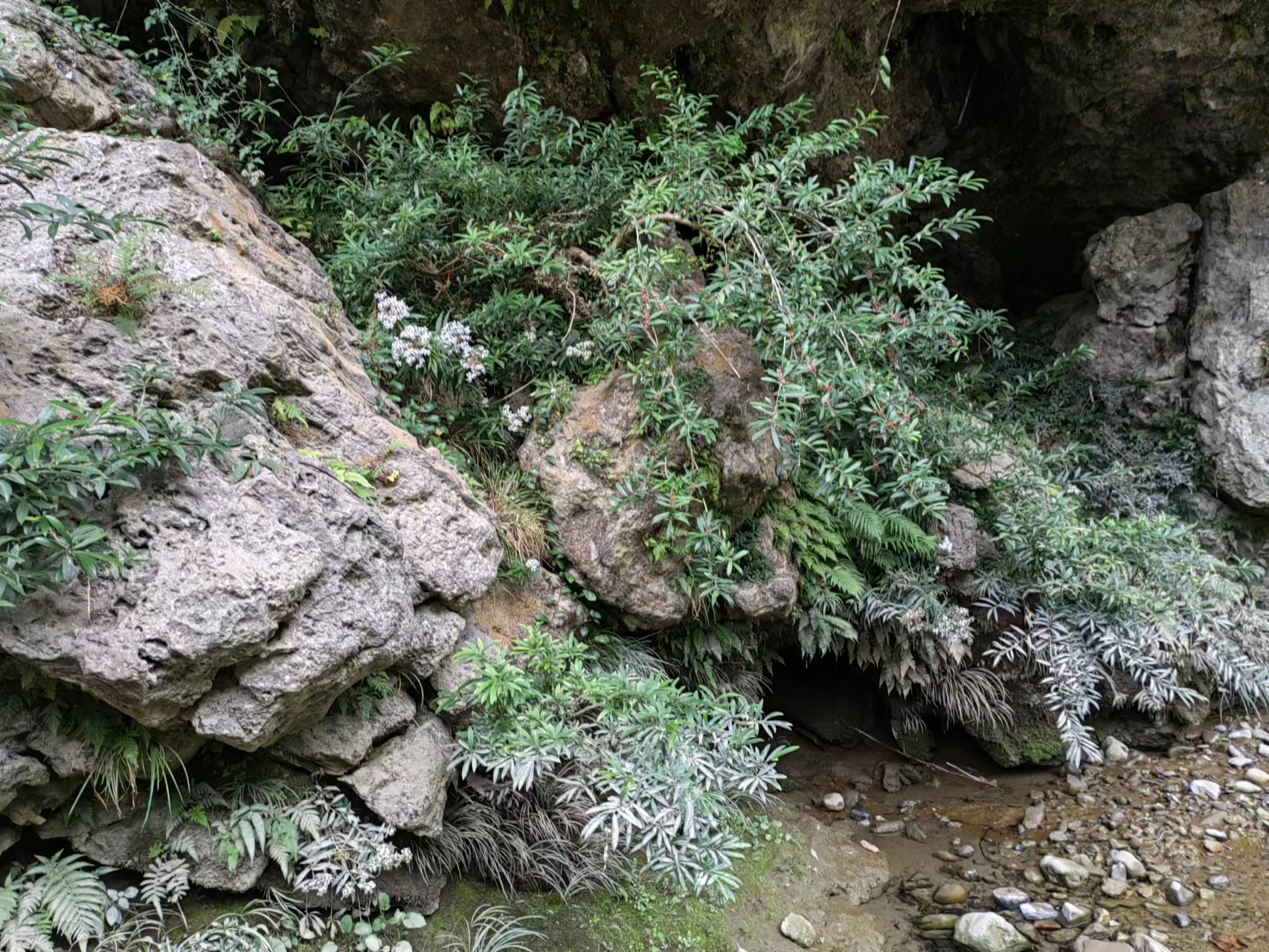 | 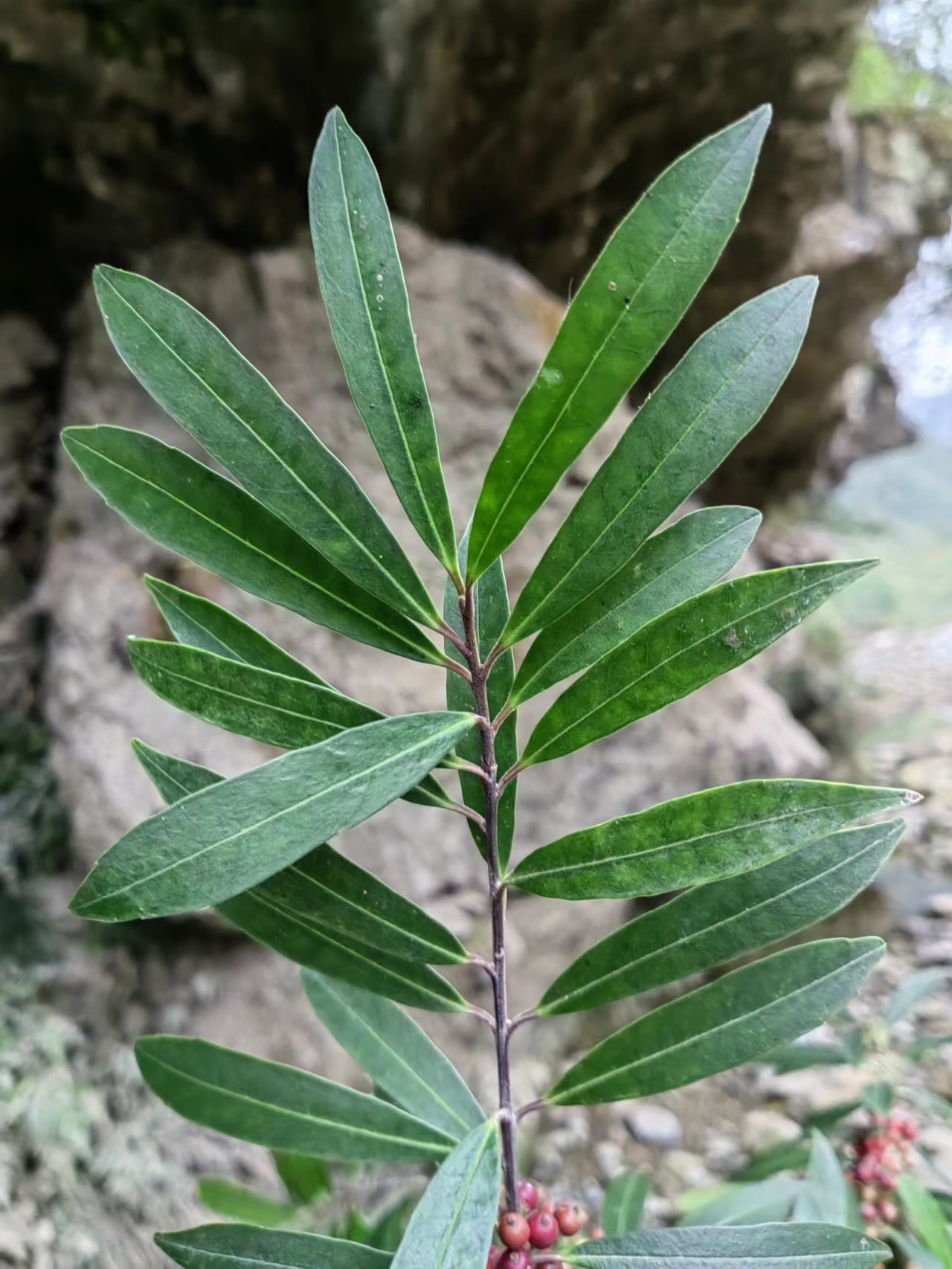 | 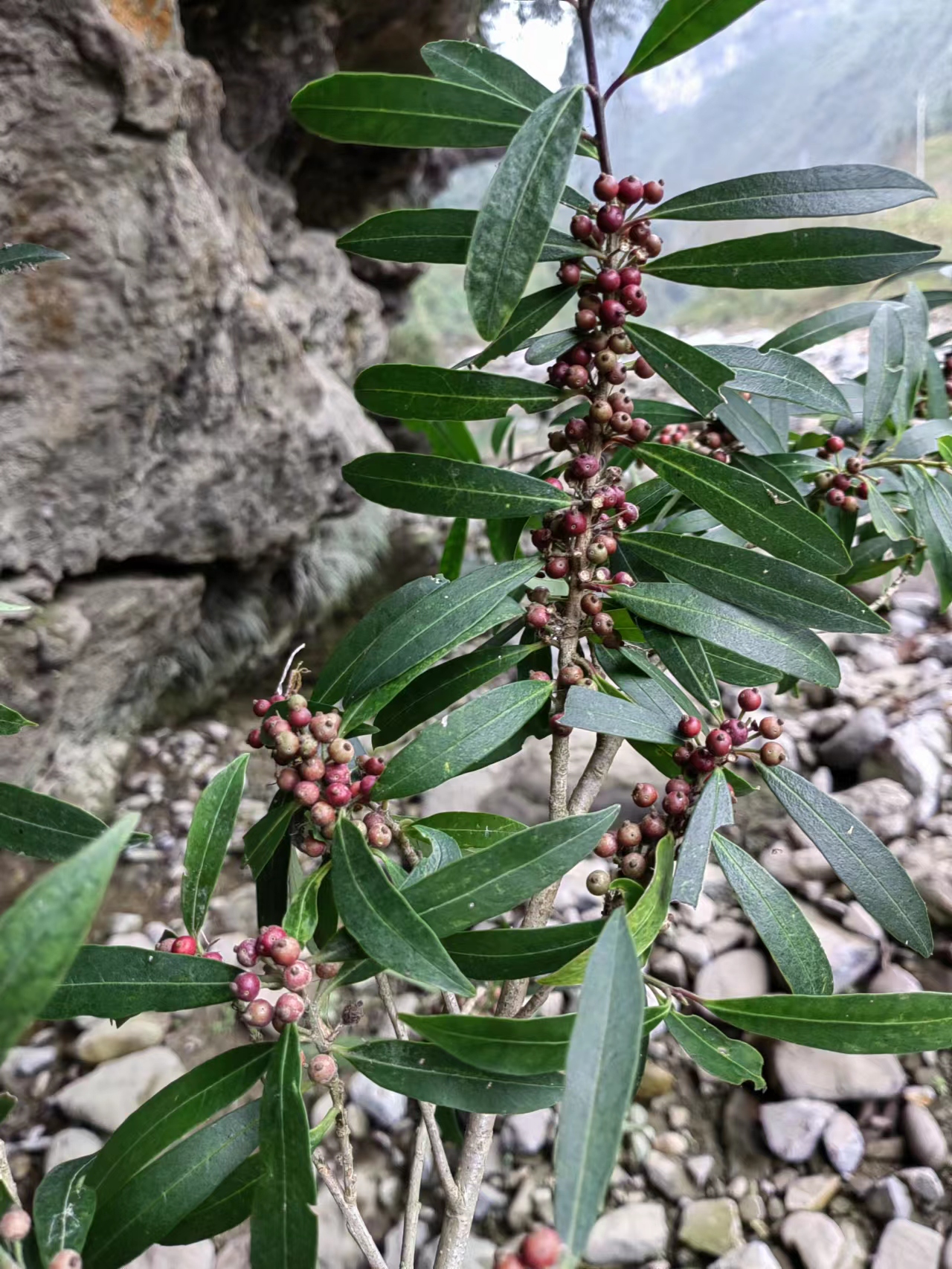 |
| --- | --- | --- |
| (**a**) | (**b**) | (**c**) |

**Figure S1** The morphological characteristics of *I. metabaptista*. **(a)** The plant. **(b)** The leaves. **(c)** The branch with fruits.
